# Hyaline cartilage differentiation of fibroblasts in regeneration and regenerative medicine

**DOI:** 10.1242/dev.200249

**Published:** 2022-01-28

**Authors:** Ling Yu, Yu-Lieh Lin, Mingquan Yan, Tao Li, Emily Y. Wu, Katherine Zimmel, Osama Qureshi, Alyssa Falck, Kirby M. Sherman, Shannon S. Huggins, Daniel Osorio Hurtado, Larry J. Suva, Dana Gaddy, James Cai, Regina Brunauer, Lindsay A. Dawson, Ken Muneoka

**Affiliations:** 1 Department of Veterinary Physiology and Pharmacology, College of Veterinary Medicine and Biomedical Sciences, Texas A&M University, College Station, TX 77843, USA; 2 Department of Hand Surgery, Union Hospital, Tongji Medical College, Huazhong University of Science and Technology, Wuhan, Hubei 430022, People's Republic of China; 3 Dewpoint Therapeutics, 6 Tide Street, Suite 300, Boston, MA 02210, USA; 4 Department of Veterinary Integrative Biosciences, College of Veterinary Medicine and Biomedical Sciences, Texas A&M University, College Station, TX 77843, USA

**Keywords:** BMP9, Articular cartilage, Digit, Fibroblasts, Joint regeneration, Regenerative medicine

## Abstract

Amputation injuries in mammals are typically non-regenerative; however, joint regeneration is stimulated by BMP9 treatment, indicating the presence of latent articular chondrocyte progenitor cells. BMP9 induces a battery of chondrogenic genes *in vivo*, and a similar response is observed in cultures of amputation wound cells. Extended cultures of BMP9-treated cells results in differentiation of hyaline cartilage, and single cell RNAseq analysis identified wound fibroblasts as BMP9 responsive. This culture model was used to identify a BMP9-responsive adult fibroblast cell line and a culture strategy was developed to engineer hyaline cartilage for engraftment into an acutely damaged joint. Transplanted hyaline cartilage survived engraftment and maintained a hyaline cartilage phenotype, but did not form mature articular cartilage. In addition, individual hypertrophic chondrocytes were identified in some samples, indicating that the acute joint injury site can promote osteogenic progression of engrafted hyaline cartilage. The findings identify fibroblasts as a cell source for engineering articular cartilage and establish a novel experimental strategy that bridges the gap between regeneration biology and regenerative medicine.

## Abstract (precis)

**Summary:** *In vivo* articular cartilage regeneration serves as a model to develop novel approaches for engineering cartilage to repair damaged joints and identifies fibroblasts as a BMP9-inducible chondroprogenitor.

## INTRODUCTION

The synovial joint is a complex multi-tissue structure with articular cartilage (AC) that covers the terminal surfaces of abutting bones. AC is composed of a highly specialized extracellular matrix (ECM) produced by articular chondrocytes (Firner et al., 2017; Luo et al., 2017) and, after maturation, AC does not turn over and displays poor regenerative capabilities; thus, damage from injury or disease is a major cause of disabilities worldwide (Medvedeva et al., 2018; Zhang et al., 2020). Cell-based engineering therapies involving expansion and differentiation of chondrocytes for transplantation (Brittberg et al., 1994) are complicated by a tendency to differentiate into fibrous cartilage and/or hypertrophic cartilage (Correa and Lietman, 2017). Mesenchymal stem cells (MSCs) from a variety of tissues are known to have chondrogenic potential and are employed as a cell source for engineering AC, although clinical success is plagued by an unstable AC phenotype (Demoor et al., 2014; Somoza et al., 2014). Promising results have been reported by following a development sequence to progressively differentiate induced pluripotent stem cells (Craft et al., 2015; Nakayama et al., 2020) or by direct differentiation of progenitor cells derived from healthy AC (Anderson et al., 2018).

In mammals, limb amputation injuries are non-regenerative; however, growth factor treatment stimulates patterned skeletal regeneration when administered during wound healing (Dawson et al., 2017; Ide, 2012; Masaki and Ide, 2007; Yu et al., 2010, 2012). Recently, BMP9 was found to stimulate regeneration of synovial joint tissues that initiate with the formation of hyaline cartilage and results in AC regeneration (Yu et al., 2019). Joint tissue regeneration is also found to result from interactions between amputated bone and intact AC in neonatal digits (Miura et al., 2020). As AC represents a non-regenerative tissue in mammals, successful regeneration indicates the presence of endogenous AC progenitor cells within the non-regenerative amputation wound. Why would non-regenerative amputation wounds contain AC progenitor cells or, indeed, any progenitor cell involved in a regeneration response? A phylogenetic analysis of regenerative capabilities among vertebrates indicates that regenerative failure among mammals evolved by modification of a primitive pro-regenerative response (Sanchez Alvarado, 2000). Successful stimulation of regeneration supports the view that cells at non-regenerative amputation injuries possess an unrealized potential to participate in a regeneration response (Dolan et al., 2018; Muneoka and Dawson, 2020). In this model of evolved regenerative failure, the fibrotic healing response involves cells with a latent potential to regenerate tissues removed by amputation. In the case of BMP9-stimulated joint regeneration, this includes progenitor cells with the potential to differentiate to articular chondrocytes. The cell types involved in fibrosis during non-regenerative amputation healing are primarily fibroblasts and immune cells (Storer et al., 2020), and as immune cells do not contribute to regenerated tissues in either amphibians or mammals (Kragl et al., 2009; Rinkevich et al., 2011), amputation wound fibroblasts represent a likely source of progenitor cells.

Cartilage regeneration does not typically occur in adult mammals, so our understanding of how different types of cartilage form comes primarily from developmental studies. The ECM produced by chondrocytes identifies the different types of cartilage found in the body; thus, expression of matrix proteins (e.g. collagens, proteoglycans and ECM-binding proteins) plays a key role in defining cartilage regeneration. Three general types of cartilage are identified: hyaline cartilage, elastic cartilage and fibrocartilage, and the appendicular skeleton develops from a hyaline cartilage template that condenses within the limb bud mesenchyme. Condensation requires the expression of *Sox9*, and collagen type II is the most prominent collagen expressed by all chondrocytes (Aigner and Stove, 2003; Bi et al., 1999). *Col2a1* cell lineage studies show that hyaline cartilage differentiates along two distinct paths: (1) endochondral ossification to form bone; and (2) AC development to form joints (Nakamura et al., 2006; Ono et al., 2014). Hyaline chondrocytes involved in endochondral ossification differentiate to hypertrophic chondrocytes and are identified as ‘transient’ hyaline cartilage, whereas AC development involves ‘permanent’ hyaline cartilage because hyaline characteristics are maintained by articular chondrocytes (Iwamoto et al., 2013). Hyaline cartilage and AC are often considered equivalent; however, AC maturation is associated with the expression of genes not expressed by hyaline cartilage, e.g. *Cilp* (Lorenzo et al., 1998). Thus, hyaline cartilage represents an embryonic precursor to AC and hypertrophic cartilage. *Prg4* cell lineage studies demonstrate that the superficial layer of AC contains stem cells that form all zonal layers of mature AC (Kozhemyakina et al., 2015; Li et al., 2017). In models of stimulated regeneration of non-regenerative digit amputation wounds, BMP2 stimulates endochondral ossification and hypertrophic chondrocyte differentiation, whereas BMP9 stimulates AC regeneration that initiates with formation of hyaline cartilage. Thus, induced regeneration displays characteristics reminiscent of cartilage formation during embryogenesis.

In this study, we investigated BMP9-induced hyaline cartilage regeneration *in vivo* and *in vitro*. Microarray analysis of BMP9-treated amputation wounds identified upregulated genes linked to both hyaline cartilage and AC differentiation. Cells of the amputation wound were cultured and found to display a parallel response to BMP9, indicating that chondroprogenitor cells can be isolated from the amputation wound. Single-cell RNAseq (scRNAseq) analysis of cultured amputation wound cells identified BMP9-responsive cells as fibroblasts. The chondrogenic response to BMP9 was used to identify an adult digit fibroblast cell line (P3 fibroblasts) (Wu et al., 2013) as a chondroprogenitor cell source, and the P3 cell line was used to develop a novel strategy to engineer hyaline cartilage. P3-BMP9 engineered hyaline cartilage was characterized and evaluated *in vivo* by implantation into an acute joint injury. Following successful engraftment, hyaline cartilage was largely stable but did not mature to AC, indicating a requirement to differentiate AC prior to transplantation. Additionally, individual hypertrophic chondrocytes of both host and graft origin were observed, suggesting that the stability of engrafted hyaline cartilage can be compromised by the injury site. These data identify fibroblasts as a novel source for hyaline cartilage regeneration, and BMP9 as a potent inducer of hyaline and articular chondrocyte differentiation. Overall, these studies establish an experimental strategy that bridges the current gap between regenerative biology and regenerative medicine of articular cartilage.

## RESULTS

### BMP9 induces amputation-derived wound cells to differentiate into chondrocytes

BMP9-stimulated joint regeneration in mice involves a chondrogenic response coupled with the formation of a synovial cavity (Yu et al., 2019). To better define this response, microarrays of induced regenerates 24 and 72 h after BMP9 treatment were generated and analyzed in comparison with control samples treated with BSA (*n*=3 for all samples). These timepoints were selected to correspond to previous *in situ* hybridization studies of the BMP9 response (Yu et al., 2019). At 24 h, 1515 unique transcripts were differentially expressed (*P*<0.05; 1.5-fold change), which included 518 upregulated and 383 downregulated annotated genes (Table S3), and 1021 unique transcripts were differentially expressed (432 upregulated, 223 downregulated genes) at 72 h (Table S4). To investigate the chondrogenic response, a list of 232 cartilage-related genes was compiled from the JAX MGI website (http://www.informatics.jax.org/mgihome/projects/aboutmgi.shtml) targeting genes associated with general cartilage development, AC and hypertrophic cartilage. Based on a literature search, this list was amended by adding 36 cartilage-related genes to generate a list of 268 cartilage-related genes, which was used to screen the two microarray datasets (Table S2).

At 24 h, 37 of the 267 chondrogenic genes were differentially expressed that included 28 upregulated and 9 downregulated genes (Table 1). The downregulated gene list included the joint development-associated gene *Osr2*. The upregulated genes included those induced during BMP9 stimulated joint regeneration (*Acan*, *Fmod*, *Prg4* and *Ucma*) (Yu et al., 2019), additional cartilage ECM genes (*Col11a1* and *Sdc3*) and AC related genes (*Chrdl2* and *Cilp*). Genes involved in BMP signaling (*Chrdl2* and *Grem1*) as well as other signaling pathways (*Fgfrl1*, *Fzd9*, *Tgfb2*, *Wif1* and *Ptger1*) were identified. A number of transcription factors (*Prrx2*, *Runx1*, *Runx2*, *Six2* and *Snai1*) were upregulated.

**Table 1.**
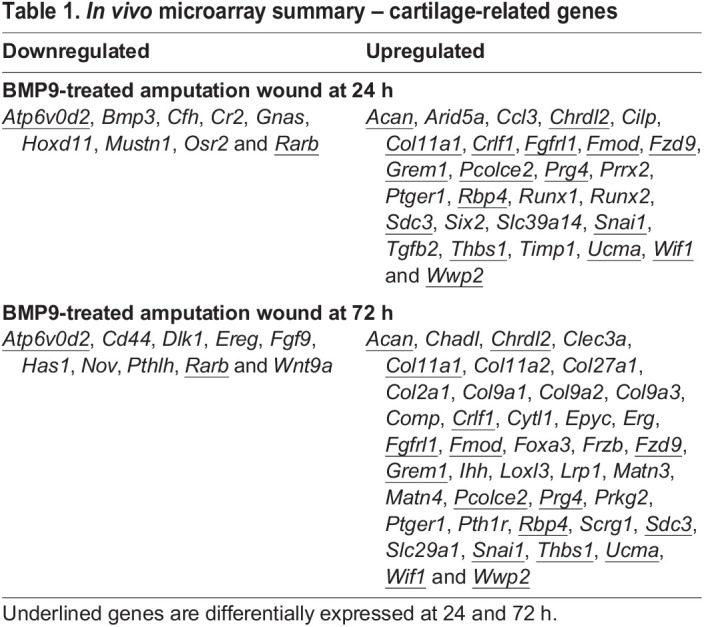
*In vivo* microarray summary – cartilage-related genes

At 72 h, 52 chondrogenic genes were differentially expressed, including 42 upregulated and 10 downregulated genes (Table 1). Two out of the 10 downregulated genes and 17 of the 42 upregulated genes were identified at the 24 h time point (Table 1), indicating maintenance of the initial BMP9 response. This list included five out of the six induced chondrogenic genes identified by *in situ* hybridization during BMP9-stimulated joint regeneration (*Ucma*, *Col2a1*, *Prg4*, *Acan* and *Fmod*) (Yu et al., 2019). In addition, the list of induced genes included major and minor collagens (*Col9a3*, *Col9a2*, *Col9a1*, *Col11a2*, *Col11a1* and *Col27a1*), collagen-binding proteins (*Matn3* and *Matn4*), proteoglycans and proteoglycan-binding proteins (*Sdc3* and *Hapln1*), and other cartilage ECM-associated proteins (*Comp*, *Scrg1* and *Chrdl2*). Genes expressed during hyaline cartilage development include *Col2a1* (Zhao et al., 1997), *Col11a1* (Yoshioka et al., 1995), *Acan* (Li et al., 2018) and *Ucma* (Surmann-Schmitt et al., 2008). Genes expressed during AC formation include *Prg4* (Kozhemyakina et al., 2015), *Chrdl2* (Nakayama et al., 2004), *Col11a2* (Lawrence et al., 2018), *Scrg1* (Ochi et al., 2006) and *Fmod* (Murphy et al., 1999). Genes linked to hypertrophic cartilage, *Col10a1* (Zheng et al., 2003), *Runx2* (Yoshida et al., 2004) and *Dlx5* (Ferrari and Kosher, 2002), were notably absent. This microarray analysis confirms previous histological and *in situ* hybridization evidence that BMP9 induces a hyaline chondrogenic response *in vivo* (Yu et al., 2019), and identifies additional BMP9 target chondrogenic genes. The data indicate that the chondrogenic response to BMP9 is rapid and progressive.

**Fig. 1.**
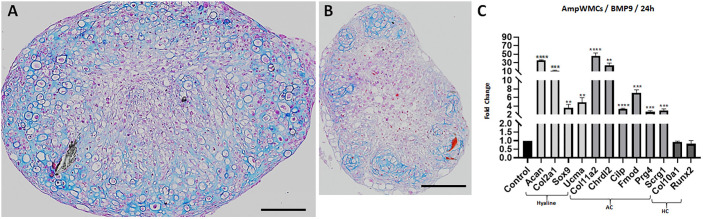
**BMP9 stimulates chondrogenesis of cultured ampWMCs.** (A-D) Chondrogenesis of ampWMCs cultures (passage 1). (A) AmpWMC centrifuged cell pellets treated with BMP9 (100 ng/ml) for 21 days (*n*=6) differentiated into cartilage with uniformly distributed chondrocytes. (B) Control untreated ampWMC pellets cultured for 21 days (*n*=3) formed isolated pockets of chondrocytes. (C) AmpWMC monolayer cultures treated with BMP9 (100 ng/ml; 24 h; *n*=3) were analyzed by qRT-PCR for expression of chondrogenic genes. Hyaline cartilage (Hyaline) and articular cartilage (AC) genes were upregulated, whereas hypertrophic cartilage (HC) genes were unaffected. Statistical analysis: parametric unpaired *t*-test in Graphpad, ****P<0.0001; ***P<0.001; **P<0.01. Scale bars: 200 µm.

To determine whether the chondrogenic response to BMP9 can be recapitulated *in vitro*, mesenchymal cells were isolated from neonatal non-regenerative digit amputations after wound closure and cultured under conditions that maintain regenerative competence (Wu et al., 2013). Amputation wound mesenchymal cells (ampWMCs) appear similar to blastema cells derived from regenerating digit tips (Lee et al., 2013) and displayed a similar limited potential for expansion in culture (Fig. S1). Thus, all experiments investigating gene expression changes associated with BMP9 treatment used cells derived from passage 1 or 2 cultures. The chondrogenic potential of ampWMCs was determined by differentiating cell pellets with or without BMP9. After 21 days of BMP9 treatment, ampWMC pellets displayed a robust response (*n*=6), forming a uniform layer of chondrocytes on the periphery of the pellet (Fig. 1A). Mallory's trichrome staining identified chondrocytes based on the presence of distinct lacunae and surrounded by Aniline Blue-positive matrix, indicative of collagen production (Fig. 1A). In contrast, control untreated ampWMC pellets (*n*=3) were smaller and contained isolated pockets of chondrocytes interspersed between non-chondrogenic cells (Fig. 1B). Control cultures indicate the presence of chondroprogenitor cells in the non-regenerative digit amputation wound, despite the absence of a chondrogenic response following digit amputation (Yu et al., 2012).

The BMP9-induced chondrogenic response of ampWMCs was analyzed by quantitative RT-PCR (qRT-PCR) in monolayer cultures focusing on chondrogenic genes identified *in vivo* (Table 1). Three different categories of chondrogenic genes were selected for analysis: (1) early chondrogenic genes associated with hyaline cartilage formation (*Sox9*, *Col2a1*, *Col11a1*, *Can* and *Ucma*); (2) AC genes (*Prg4*, *Chrld2*, *Fmod*, *Scrg1*, *Cilp* and *Col11a2*); and (3) hypertrophic cartilage genes (*Col10a1* and *Runx2*). Compared with untreated controls, 24 h of BMP9 stimulation results in enhanced transcript levels of hyaline cartilage and AC genes, but not hypertrophic cartilage genes (Fig. 1C). The possibility that the BMP9 response resulted solely from stimulated proliferation of endogenous chondroprogenitor cells cannot explain the level of enhanced gene expression within 24 h, thus indicating that BMP9 induces chondrogenic gene expression. These studies indicate that (1) chondroprogenitor cells are present in the healing non-regenerative digit amputation wound, (2) BMP9 induces chondrogenesis of ampWMCs and (3) BMP9-stimulated chondrogenic regeneration *in vivo* can be replicated *in vitro*.

### Amputation-derived mesenchymal wound cells are fibroblasts

**Fig. 2.**
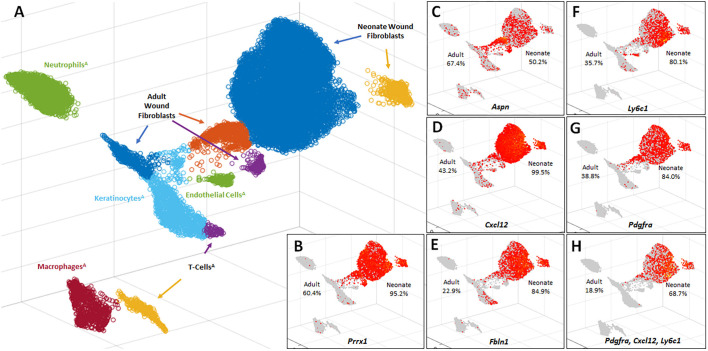
**scRNAseq analysis of ampWMCs.** (A) Uniform manifold approximation and projection (UMAP) plot of 7568 adult digit amputation wound cells (Storer et al., 2020) and 13,470 neonatal ampWMCs. Each circle represents a single cell and cells assigned to the same cluster are similarly colored. Cell-type identities were assigned using the scGEAToolbox. Neonatal and adult (^A^) amputation wound cells are distinct and do not overlap. UMAP identifies ampWMCs as fibroblasts that form two distinct neonatal clusters and three clusters of adult wound fibroblasts. (B) UMAP overlay identifying cells expressing *Prrx1*, a limb-specific fibroblast marker gene. (C-G) UMAP overlays identifying cells expressing key fibroblast marker genes from different adult tissues (Buechler et al., 2021): *Aspn* (C), *Cxcl12* (C), *Fbln1* (D), *Ly6c1* (F) and *Pdgfra* (E). (H) UMAP overlay identifying cells co-expressing *Pdgfra*, *Cxcl12* and *Ly6c1*. Each red circle identifies a cell expressing the gene(s) of interest; gray regions identify non-expressing cells. The frequency of adult and neonatal cell expression is indicated on the left and right side of each UMAP plot, respectively.

These results led to the question of which cell type in the amputation wound responds to BMP9. To characterize BMP9-responsive cells, passage 1 ampWMCs were collected for scRNAseq analysis. The neonatal ampWMC scRNAseq dataset included transcriptomes from 13,474 cells and was analyzed using scGEAToolbox (Cai, 2019; Osorio et al., 2020) to determine cell type. Cell type determination used the PanglaoDB database of cell-type marker genes derived from published mouse scRNAseq studies (Franzen et al., 2019). The ampWMC dataset was analyzed with published amputation wound datasets for comparative analysis. Direct comparisons with published scRNAseq datasets of adult P2 level digit amputation wound cell transcriptomes (Storer et al., 2020) was carried out by establishing a combined dataset that included the ampWMC dataset with this published dataset. Adult amputation wound cell datasets were pooled from 10- and 14-days post-amputation digits, which consisted of transcriptomes of 7658 cells, of which 1654 cells were identified as fibroblasts. The combined digit amputation dataset included 21,132 total cell transcriptomes (7658 adult cells and 13,474 neonatal ampWMCs). UMAP plots of this combined dataset identified macrophage, neutrophil, T-cell, keratinocyte, endothelial cell and three distinct fibroblast clusters derived from the adult dataset, whereas the cultured neonatal ampWMCs formed one large and one small fibroblast cluster (Fig. 2A). The adult and neonatal fibroblast clusters were non-overlapping. The analysis of ampWMCs identified the vast majority of cells as fibroblasts, and this conclusion was supported by a high frequency of cells expressing the limb-specific fibroblast marker genes *Prrx1* (95.06%) (Fig. 2B) coupled with a paucity of cells expressing marker genes for other cell types known to be present at non-regenerative digit amputation wounds: epidermis (*Krt14*, 0.10%), bone (*Bglap*, 0.71%), endothelial cells (*Pecam1*, 0.27%), Schwann cells (*Plp1*, 1.94%), monocytes (*Lyz2*, 1.76%), vascular smooth muscle cells (*Rgs5*, 0.63%) and T cells (*Cd3g*, 0.00%) (Johnson et al., 2020; Storer et al., 2020).

To further confirm the fibroblast identity of ampWMCs, the combined dataset was analyzed for expression of 23 key fibroblast marker genes identified from a whole-body adult fibroblast scRNAseq atlas (Buechler et al., 2021). A dual analysis of differential gene expression coupled with the percentage of cells expressing key fibroblast marker genes was carried out for adult and neonatal wound fibroblasts (15,074 total cells) using the scGEAToolbox. Differential expression analysis identified 10 key fibroblast marker genes expressed at higher levels in adult wound fibroblasts compared with neonatal wound fibroblasts, four genes not differentially expressed and nine genes expressed at higher levels in neonatal fibroblasts (Table 2). The top four differentially expressed genes based on expression frequency for adult wound fibroblasts are *Aspn* (67.4%), *Cxcl12* (43.2%), *Pdgfra* (38,8%) and *Ly6c1* (35.7%), and, of these genes, only *Aspn* was expressed at a higher level in adult wound fibroblasts compared to neonatal fibroblasts (Fig. 2B-D,F,G). The top four differentially expressed genes based on expression frequency for neonatal wound fibroblasts are *Cxcl12* (99.4%), *Fbln1* (84.7%), *Pdgfra* (83.7%) and *Ly6c1* (80.6%), and all of these genes are enriched in neonatal fibroblasts compared with adult fibroblasts (Fig. 2D-G). It is noteworthy that three out of the four fibroblast marker genes expressed at high frequencies in neonatal and adult wound fibroblast populations are overlapping (*Cxcl12*, *Pdgfra* and *Ly6c1*), suggesting that these developmentally distinct digit fibroblast populations are related. The high frequency of fibroblasts expressing all three genes, especially in neonatal fibroblasts, suggests that individual fibroblasts are co-expressing multiple key tissue-specific fibroblast genes. Indeed, 68.7% of neonatal amputation wound fibroblasts co-express all three fibroblast marker genes, whereas the level of co-expression in adult amputation fibroblasts was found to be 18.9% (Fig. 2H). These results demonstrate that neonatal ampWMCs are fibroblasts and that these cells possess latent chondroprogenitor cell characteristics that can be activated by BMP9. As neonatal and adult amputations display a similar chondrogenic response to BMP9 *in vivo* (Yu et al., 2019) and fibroblasts are the predominant non-inflammatory mesenchymal cell type present at the amputation wound (Storer et al., 2020), the results support the conclusion that BMP9 stimulates chondrogenesis of fibroblasts involved in non-regenerative healing of digit amputation wounds.

**Table 2.**
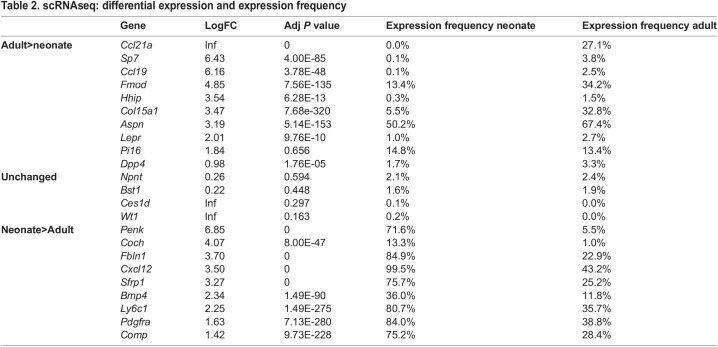
scRNAseq: differential expression and expression frequency

### BMP9 induces digit fibroblasts to differentiate into articular chondrocytes

**Fig. 3.**
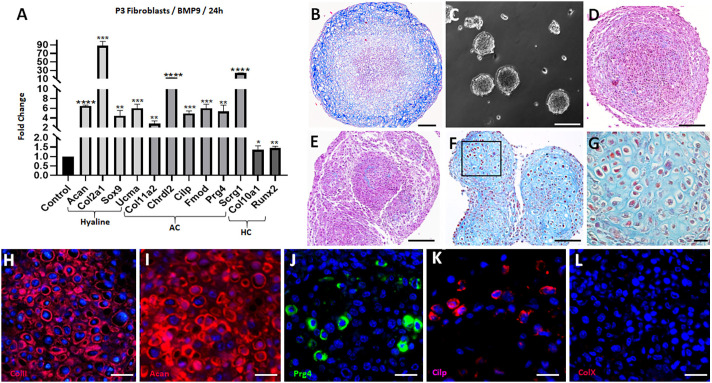
**P3-BMP9 cultures differentiate hyaline cartilage.** (A) P3 monolayer cultures treated with BMP9 (100 ng/ml; 24 h; *n*=3) and analyzed by qRT-PCR for chondrogenic gene expression. Hyaline cartilage (Hyaline) and articular cartilage (AC) genes were upregulated, whereas hypertrophic cartilage (HC) genes were largely unaffected. Statistical analysis: parametric unpaired *t*-test in Graphpad, ****P<0.0001; ***P<0.001; **P<0.01; *P<0.05. (B) P3-BMP9 pellet cultures (21 days; *n*=2) contain differentiated chondrocytes along the periphery but central cells are necrotic. (C) P3 SA cultures form cell aggregates after 4 days. (D) P3 control SA cultures (36 days; *n*=6) enlarge to a size similar to pellet cultures, with no evidence of central necrosis. (E) P3-BMP2 SA cultures (100 ng/ml; 36 days; *n*=2) fail to differentiate chondrocytes. (F) P3-BMP9 SA cultures (100 ng/ml; 36 days; *n*=6) differentiate hyaline cartilage. (G) High magnification of BMP9-stimulated hyaline cartilage showing chondrocyte doublets surrounded by a collagen rich matrix. (H-L) P3-BMP9 SA cultures (100 ng/ml; 36-days; *n*=2) immunostained for chondrogenic markers. (H,I) Hyaline cartilage markers ColII (H) and Acan (I) are expressed by the majority of cells. (J,K) AC markers Prg4 (J) and Cilp (K) are expressed by cells scattered throughout the cartilage. (L) ColX is not expressed by any cells. Scale bars: 200 µm in B; 100 µm in C-F; 20 µm in G; 25 µm in H-L.

Fibroblasts isolated from the unamputated terminal phalangeal element (P3 fibroblasts) can be expanded in culture while retaining position-specific characteristics and regenerative competence (Wu et al., 2013). We tested the BMP9 response of P3 fibroblasts using our chondrogenesis assays. Monolayer cultures of P3 fibroblasts were induced by BMP9 to upregulated hyaline cartilage and AC genes, while transcripts of hypertrophic cartilage genes were minimally changed (Fig. 3A). Thus, both neonate ampWMCs (Fig. 1C) and adult P3 fibroblasts display a rapid chondrogenic response to BMP9 that is directed towards hyaline cartilage and AC but not towards hypertrophic cartilage.

MSCs are known precursors for bone, cartilage and adipose tissue (Dominici et al., 2006), and, as P3 fibroblasts are heterogenous, they likely contain MSCs. BMP9-treated P3 fibroblast cell pellets differentiated into chondrocytes, indicating chondrogenic potential (Fig. 3B); however, P3 fibroblasts failed to differentiate all other MSC phenotypes when tested using commercially available assays (Fig. S2A-D) and did not display a cell surface phenotype characteristic of MSCs, based on flow cytometry (Fig. S2E,F); thus, P3 fibroblasts cannot be characterized as MSCs (Dominici et al., 2006).

Cultures of centrifuged P3 fibroblast pellets displayed BMP9-stimulated chondrogenesis of peripheral cells with extensive necrosis in the central region (Fig. 3B), and this warranted the development of an alternative approach to differentiate cartilage. A 4-day self-aggregation (SA) protocol in Petri dishes was established to promote formation of cell clusters prior to BMP9 treatment (Fig. 3C). Untreated SA cultures enlarged to form aggregates that approximate the size of cell pellets and, after 36 days, histological analysis indicated healthy undifferentiated cells and an absence of necrosis (Fig. 3D). SA cultures treated with BMP2 (100 ng/ml) also failed to stimulate a chondrogenic response (Fig. 3E), whereas SA cultures treated with BMP9 (100 ng/ml) formed large cartilage tissue networks that were histologically indistinguishable from hyaline cartilage (Fig. 3F,G). Immunostaining studies of P3 BMP9-treated aggregates indicate a high level of Col II (Fig. 3H) and aggrecan expression (Fig. 3I), confirming chondrocyte differentiation. In addition, cells expressing AC markers, Prg4 (Fig. 3J) and Cilp (Fig. 3K) were scattered throughout the cartilage, indicating differentiation of articular chondrocytes. Cells expressing Col X were absent, indicating that hypertrophic chondrocytes did not differentiate (Fig. 3L). Control untreated P3 fibroblast aggregates cultured for 36 days were immuno-negative for ColII, Acan, Prg4, Cilp and ColX (Fig. S3).

**Fig. 4.**
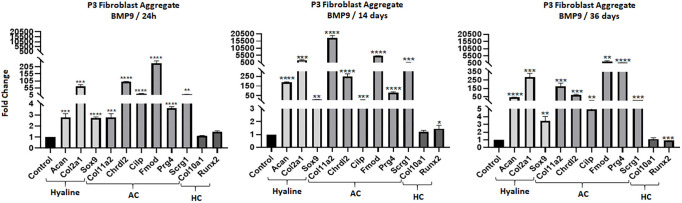
**BMP9 stimulates chondrogenic gene expression in SA cultures.** qRT-PCR analysis of P3-BMP9 SA cultures treated with BMP9 for 1 (left), 14 (middle) and 36 (right) days. At all timepoints, hyaline cartilage (Hyaline) and articular cartilage (AC) genes were upregulated, whereas hypertrophic cartilage (HC) genes were largely unaffected. Statistical analysis: parametric unpaired *t*-test in Graphpad, ****P<0.0001; ***P<0.001; **P<0.01; *P<0.05.

The temporal response of P3-BMP9 treated aggregates was analyzed by qRT-PCR for expression of chondrocyte transcripts after different culture times (1, 14 and 36 days). 1 day after BMP9 treatment, hyaline cartilage and AC genes were upregulated, whereas hypertrophic cartilage genes were not (Fig. 4A). This chondrogenic response was similar to that of monolayer cultures of ampWMCs (Fig. 1C) and P3 fibroblasts (Fig. 3A), and indicates that culture conditions (i.e. 2D versus 3D) do not modify the P3 fibroblast response. At later timepoints, this expression profile was qualitatively similar but differed quantitatively (Fig. 3B,C). All of the hyaline cartilage genes displayed their highest relative level of expression at 14 days, with transcript levels declining by 36 days, and most of the AC genes displayed a similar pattern with the exception of *Prg4*, which showed a continuous increase in transcript levels during the 36-day timeline. Hypertrophic cartilage transcripts (*Col10a1* and *Runx2*) were largely unaffected by BMP9 treatment at all timepoints analyzed.

The similarity of the chondrogenic responses between ampWMCs and P3 fibroblasts suggests that the two are related. To explore this hypothesis, microarray analysis of P3-BMP9 treated aggregates was performed after 3 days of treatment and compared with untreated control aggregates. A total of 6016 differentially expressed transcripts were identified when compared to untreated controls (*n*=3) (Table S5; *P*<0.05; 1.5-fold change). This included 2428 upregulated and 3177 downregulated transcripts. An analysis of differentially expressed cartilage-related genes (Table S2) identified 109 (39 downregulated and 71 upregulated) genes (Table 3). The list of downregulated genes included genes associated with joint development (*Ors1*, *Ors2* and *Cd44*), developmentally important transcription factors (*Hoxb3*, *Hoxd3* and *Scx*) and signaling pathways (*Bmp2*, *Bmp4*, *Bmp6*, *Fgf18*, *Pthlh*, *Rarb*, *Smad3*, *Tgfbr2*, *Wnt7a*, *Wnt7b* and *Wnt9a*). Eight of the 39 downregulated chondrogenic genes were also downregulated by BMP9 in *in vivo* microarrays, and there were four ambiguous genes that were downregulated by BMP9 in P3 BMP9-treated aggregates but upregulated by BMP9 *in vivo* (*Ccl3*, *Chadl*, *Runx1* and *Thbs1*) (Table 3). The list of 71 upregulated chondrogenic genes was remarkable because 43.7% of the genes (31/71) overlapped with BMP9-induced genes *in vivo* (Table 3). Many of the genes are associated with hyaline cartilage and AC, but genes associated with hypertrophic cartilage were notably absent. Overall, the data show that P3 fibroblasts and ampWMCs respond similarly to BMP9 by differentiating into hyaline cartilage that contains articular chondrocytes.

**Table 3.**
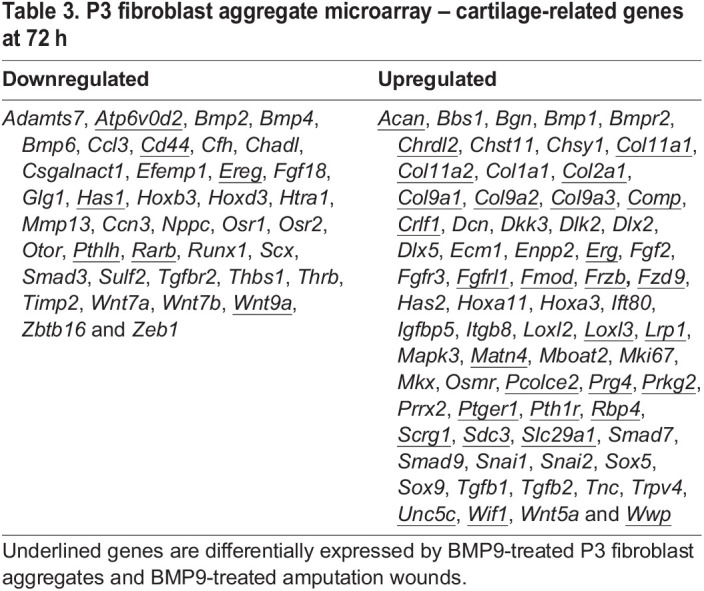
P3 fibroblast aggregate microarray – cartilage-related genes at 72 h

Amputation wound fibroblasts and P3 fibroblasts both display a similar chondrogenic response to BMP9, yet wound fibroblasts are derived from non-regenerative digit amputations, whereas P3 fibroblasts are expanded from uninjured digit tissue. The role of wound healing in the BMP9 response was investigated by implanting a BMP9 bead into an uninjured adult digit to determine whether uninjured fibroblasts displayed a chondrogenic response *in vivo*. In contrast to the response of wound fibroblasts in neonates and adults that display a robust chondrogenic response to BMP9 (Yu et al., 2019), no chondrogenic response was observed by fibroblasts of uninjured digits (Fig. S4). These results suggest that digit fibroblasts acquire chondroprogenitor characteristics during the process of amputation injury healing *in vivo*, and also by enzymatic dissociation and expansion in a two-dimensional cell culture environment.

### Chondrogenic stability of P3-BMP9 engineered hyaline cartilage

**Fig. 5.**
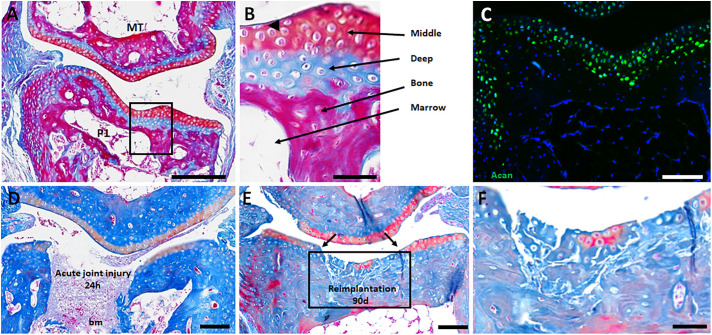
**Metatarsal-phalangeal (MtP) joint defect.** (A,B) Abutting AC surfaces each display a layered organization consisting of a middle layer and a deep layer (*n*=2). A single flattened superficial cell is shown in B (arrowhead) but these cells are infrequent and do not form a continuous cell layer. (C) Articular chondrocytes of the middle and deep layers are characterized by the expression of Acan (*n*=3). (D) An acute defect of the phalangeal surface removes the AC and underlying bone, exposing the joint to the bone marrow (bm). 24 h after injury, the acute defect is filled with cells contiguous with the bone marrow (*n*=4). (E,F) After 3 months, re-engrafted excised AC contained cells that retained staining characteristics of articular chondrocytes (*n*=3). Scale bars: 200 µm in A; 40 µm in B; 100 µm in C-E; 50 µm in F.

The cell culture model described here can serve as a foundation to engineer AC for joint repair. To investigate *in vivo* stability of engineered cartilage, we established an acute joint defect model in the metatarsal-phalangeal (MtP) joint of immunodeficient (NOD/Scid) host mice. The MtP joint consists of the distal end of the metatarsus (Mt) and the proximal end of the first phalangeal (P1) element (Fig. 5A). Abutting AC surfaces are histologically similar: each consists of two prominent zones that are identified as middle and deep (Fig. 5B). Scattered flattened cells are observed on the AC surface but these cells do not form a contiguous superficial cell layer and are not immunopositive for Prg4. Subchondral bone separates the AC layers from the bone marrow. Acan immunopositive cells are specific to both middle and deep layers, and identify the AC (Fig. 5C). A defect in the P1 AC is created surgically by removing the central segment and subchondral bone, thereby exposing the joint cavity to the bone marrow (Fig. 5D). Control joint defects in which excised tissue was immediately transplanted back into the defect demonstrate that implanted tissue survives and retains a differentiated phenotype after a 90-day engraftment period (Fig. 5E,F).

**Fig. 6.**
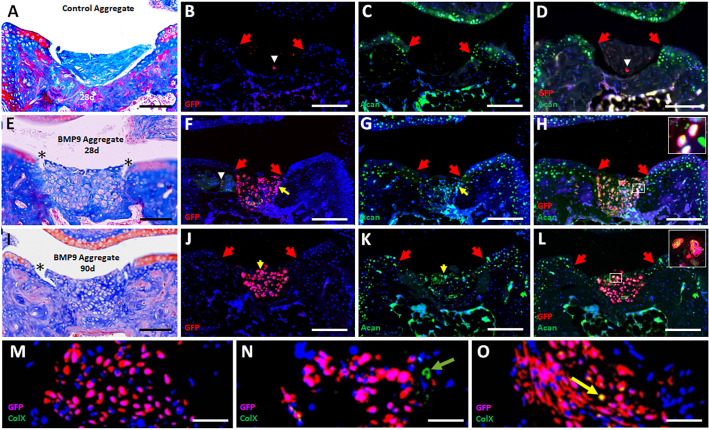
**Engraftment of P3-BMP9 engineered hyaline cartilage into the MtP joint defect.** Red arrows identify the boundary of the injury in immunostained sections in B-D,F-H,J-L. (A-D) Engrafted control tissue from untreated P3 SA cultures (36 days) after 28 days. (A) Histological assessment: engrafted tissue appears acellular and not integrated with host tissues. (B) GFP immunostaining identified few positive cells (white arrowhead) associated with the implant. (C) Acan immunostaining indicates the implanted tissue is devoid of Acan-positive cells. (D) Overlay of GFP and Acan immunostaining shows that GFP-positive cells (white arrowhead) are negative for Acan. (E-H) Hyaline cartilage implants from P3-BMP9 SA cultures (36 days; *n*=6) analyzed after 28 days. (E) Histological assessment: the MtP defect contains cartilage tissue that is tightly adherent to surrounding bone tissue but not adherent to surface AC (asterisk). (F) GFP immunostaining is localized to the joint defect with a few GFP-positive cells (white arrowhead) invading neighboring host tissue. (G) Acan immunostaining identifies immunopositive chondrocytes within the implanted tissue. (H) Overlay of GFP and Acan immunostaining demonstrates Acan expression by implanted cells. Inset in H shows a double-labeled cell cluster at higher magnification. Yellow arrows in F and G identify the cell cluster shown at higher magnification. (I-L) Hyaline cartilage implants from P3-BMP9 SA cultures (36 days; *n*=12) analyzed after 90 days. (I) Histological assessment: the MtP defect contains cartilage tissue that is tightly adherent to surrounding bone tissue but not adherent to surface AC (asterisk). (J) GFP immunostaining is localized to the joint defect. (K) Immunostaining identifies Acan-positive cells spanning the joint defect. (L) Overlay of GFP and Acan immunostaining shows that implanted cells maintain expression of Acan. Inset shows a double-labeled cell cluster at high resolution. Yellow arrows in J and K identify the cell cluster shown at higher magnification. (M-O) Double immunostaining for GFP and ColX. (M) ColX-positive cells were not found in four out of 12 samples. (N) ColX-positive/GFP-negative cells (green arrow) were found in five out of 12 samples. (O) ColX-positive/GFP-positive cells (yellow arrow) were found in five out of 12 samples. Scale bars: 100 µm in A,E,I; 200 µm in B-D,F-H,J-L; 50 µm in M-O.

The stability of P3-BMP9 engineered hyaline cartilage was tested by implantation into the joint defect. Hyaline cartilage was engineered using *Gfp*-expressing P3 fibroblasts (*Gfp*-P3) and after 36 days in culture the resulting tissue was engrafted into the joint defect. Histological and immunohistochemical analyses (GFP, Acan and ColX) after 28 and 90 days determined implant survival, integration with host tissues and retention of cartilage characteristics. Control implants engineered without BMP9 treatment (*n*=7) (see Fig. 3D and Fig. S3) did not form cartilage after implantation (Fig. 6A) but filled the defect with matrix that was largely acellular (Fig. 6B-D), indicating that cell survival was compromised.

Implants of P3-BMP9 engineered hyaline cartilage analyzed at 28 days (*n*=6) and 90 days (*n*=12) were equivocal. All samples survived and maintained a hyaline chondrogenic phenotype, but no samples were found to completely integrate with host tissues (Fig. 6E,I). Implanted cartilage adhered tightly to host bone tissue, but not with host AC where gaps between host and implanted cartilage are apparent. GFP immunostaining confirmed implanted cartilage survival and demonstrated continuity within the implant as well as sharp boundaries with unlabeled host cells (Fig. 6F,J). Overall, there was little evidence of cell invasion into host tissues or vice versa, although individual GFP-positive cells were occasionally observed in neighboring host tissue (Fig. 6F). Immunostaining identified Acan expression by chondrocytes throughout the implanted tissue, indicating that engrafted cells maintained chondrogenic characteristics (Fig. 6G,K). Double immunostaining for GFP and Acan identified numerous double-labeled cells within the engrafted tissue, confirming that many implanted cells retained a chondrogenic phenotype, although the level of co-expression was not 100% (Fig. 6H,L). These studies indicate that the chondrogenic phenotype of BMP9 engineered hyaline cartilage from P3 fibroblast aggregates was maintained following engraftment into an acute joint defect.

A major concern of AC engineering is that implanted cartilage can undergo endochondral ossification leading to pathological osteogenesis (Ripmeester et al., 2018). To determine whether pathological progression of engrafted tissue or surrounding host tissue occurred, 90-day samples were analyzed for ColX expression to determine the presence of hypertrophic chondrocytes. Hypertrophic chondrocytes are not observed in the intact MtP joint or in engineered hyaline cartilage prior to transplantation, so their presence following engraftment would suggest *in vivo* conditions that promoted pathological osteogenesis. Hypertrophic chondrocytes were not found in four out of the 12 (33.3%) samples (Fig. 6M), but eight samples (66.6%) contained individual hypertrophic chondrocytes. Three out of the eight positive samples contained only host-derived hypertrophic chondrocytes (Fig. 6N), three samples contained only graft-derived cells (Fig. 6O) and two samples contained both host- and graft-derived cells. The presence of graft-derived hypertrophic chondrocytes indicated that P3-BMP9 engineered hyaline cartilage maintains a potential for hypertrophic chondrocyte differentiation. To test this, P3-BMP9 engineered hyaline cartilage (36-day cultures) was cultured an additional 20 days in BMP2 (P3-BMP9-BMP2) or, as a control, in BMP9. Control P3-BMP9 cultures (56 days) maintained a hyaline cartilage phenotype that was confirmed based on histology, qRT-PCR and ColX immunostaining (Fig. S5A,B,D), whereas P3-BMP9-BMP2 cultures were found to contain numerous ColX-positive hypertrophic chondrocytes (Fig. S5C). These studies indicate that P3-BMP9 engineered hyaline cartilage retain a potential for hypertrophic chondrocyte differentiation and suggest that the acute joint defect can promote pathological osteogenesis of engrafted hyaline cartilage. These engraftment studies indicate that the differentiative state of engineered AC prior to engraftment will be important to minimize post engraftment osteogenesis.

## DISCUSSION

Endogenous joint repair and AC regeneration in mammals does not readily occur so the demonstration that BMP9 stimulates joint and AC regeneration at a non-regenerative amputation wound in mice warrants further mechanistic investigation. There are two distinct outcomes from these studies. The first involves the biological response to BMP9 as a regeneration-inducing agent *in vivo*; the second involves exploration of a regenerative medicine strategy aimed at engineering AC. Although regeneration biology and regenerative medicine share a common interest, i.e. regeneration, these two fields remain largely separate. Regeneration biology is focused on models that display a high level of endogenous regenerative capabilities, e.g. invertebrates, fish and amphibians, whereas regenerative medicine is focused on mammals, particularly humans, that display relatively poor regenerative capabilities. The development of mammalian models of endogenous regenerative responses (Seifert and Muneoka, 2018) and the demonstration that regeneration can be stimulated at non-regenerative injury sites in mice (Dawson et al., 2017; Han et al., 2003; Ide, 2012; Masaki and Ide, 2007; Yu et al., 2019, 2010, 2012) provide an opportunity to bridge these two disparate but related fields. By focusing on BMP9-stimulated AC regeneration *in vivo* and *in vitro*, we have established a novel strategy for investigating regeneration and regenerative failure in mammals that can impact therapeutic strategies for regenerative medicine.

### Regeneration biology

Following amputation, the mammalian digit is non-regenerative, with the exception that the digit tip possesses endogenous regenerative ability (Muneoka et al., 2008; Simkin et al., 2015; Storer and Miller, 2020). This regenerative response in mice represents a rare example of blastema-mediated epimorphic regeneration in mammals. Canonical BMP signaling is required for regeneration (Han et al., 2003; Yu et al., 2010) but, surprisingly, BMP9 treatment also inhibits digit tip regeneration by precociously stimulating *Vegfa*-mediated angiogenesis during blastema formation (Yu et al., 2014). Alternatively, BMP9 stimulates joint regeneration at non-regenerative amputation wounds but this response is not associated with enhanced angiogenesis (Yu et al., 2019) or with enhanced *Vegfa* expression (see Tables S3 and S4). Stimulation of non-regenerative amputation wounds by BMP9 or BMP2 (Yu et al., 2012) directly stimulates chondroprogenitor cells and does not involve blastema formation, so amputation level-dependent BMP9-mediated effects reflect differences in BMP9-responsive cells present at regenerating versus non-regenerative wounds. Supporting this conclusion, BMP9 either enhances or inhibits angiogenesis in different experimental model systems (Scharpfenecker et al., 2007; Xiao et al., 2020), and is known to display a context-dependent response on vascular development (Chen et al., 2013). Thus, the stimulatory and inhibitory effects of BMP9 in different regeneration models can be explained by its role in regulating angiogenesis during blastema formation, on the one hand, and in stimulating chondroprogenitor cells, on the other.

Amputation at the level of the second phalanx is a model of fibrotic healing and regenerative failure that can be stimulated to regenerate (Dawson et al., 2016; Turner et al., 2010; Yu et al., 2012). Treatment of the amputation wound with BMP2 or BMP7 stimulates skeletal regeneration by endochondral ossification where differentiating hypertrophic chondrocytes mediate the regeneration of new bone (Dawson et al., 2017; Yu et al., 2010, 2012). Alternatively, BMP9 stimulates joint regeneration, inducing ectopic hyaline cartilage coupled with a synovial cavity that articulates with the stump bone (Yu et al., 2019). Both responses initiate with induced early chondrogenic genes (i.e. *Col2a1* and *Sox9*) but with distinct outcomes; hypertrophic cartilage precedes skeletal regeneration, whereas hyaline cartilage precedes AC regeneration. BMP9 stimulates a rapid upregulation of genes specifically associated with AC and not hypertrophic cartilage, indicating that BMP9 directs chondroprogenitor cell differentiation toward articular chondrocytes and not towards hypertrophic chondrocytes. It is noteworthy that hypertrophic chondrocytes can be induced in P3-BMP9 engineered hyaline cartilage *in vivo* and *in vitro* following treatment with BMP2, indicating a differentiation potential that is actively inhibited by BMP9. Thus, BMP9 represents an inducer of AC differentiation during joint regeneration.

The cells responding to BMP9 are the fibroblasts of the amputation wound. P2 amputation undergoes non-regenerative wound healing, and fibroblasts are the principal mesenchymal cell type involved in the response (Storer et al., 2020). Amputation wound fibroblasts are stimulated by BMP9 to initiate a chondrogenic program that results in the differentiation of hyaline cartilage *in vivo* and *in vitro*. Fibroblast re-programming into chondrocytes has been demonstrated by ectopic gene expression (Hiramatsu et al., 2011), indicating chondrogenic potential. In amphibian limb regeneration, amputation wound fibroblasts over-contribute to blastema formation and undergo chondrogenesis to form the hyaline cartilage anlagen of the regenerating limb (Dunis and Namenwirth, 1977; Kragl et al., 2009; Muneoka et al., 1986). Thus, the presence of wound fibroblasts at non-regenerating mammalian amputations with chondroprogenitor characteristics is predicted based on an evolved regenerative failure model (Muneoka and Dawson, 2020; Sanchez Alvarado, 2000). How chondroprogenitor fibroblasts arise at the mammalian amputation wound remains unclear as chondrogenesis is not stimulated by treatment of uninjured or immediately amputated digits with either BMP9 (Fig. S4) or BMP2 (Dawson et al., 2017). One explanation is that fibroblasts respond to amputation injury by dedifferentiation to a developmentally immature phenotype, thereby acquiring a chondroprogenitor phenotype (Gerber et al., 2018). In mammals, this idea is supported by the demonstration that non-regenerative fibroblasts can participate in regeneration and display epithelial-mesenchymal signaling characteristics reminiscent of early development (Wu et al., 2013).

### Regenerative medicine

The successful stimulation of AC regeneration *in vivo* provides the impetus to explore the potential for translational repair in humans. A clinically successful approach to engineering AC is a complex problem that includes: (1) identification of a cell source, (2) establishment of a differentiation protocol and (3) post-transplantation assessment of tissue survival, integration and maintenance of differentiation (Iwamoto et al., 2013). Most approaches to engineering AC use stem cells because they represent a plentiful cell source and a differentiation protocol involving high density cell cultures treated with TGF (Correa and Lietman, 2017). Unfortunately, a common clinical outcome of implanted cartilage engineered using this approach is a transient repair response, with chondrocytes eventually differentiating into fibrocartilage or hypertrophic cartilage (Demoor et al., 2014; Somoza et al., 2014). The current study establishes a completely novel approach to AC engineering that exploits the chondrogenic response of an *in vivo* joint regenerative response (Yu et al., 2019). Fibroblasts of the amputation wound represent a BMP9-responsive chondroprogenitor cell type, and the P3 fibroblast cell line (Wu et al., 2013) displays an analogous chondrogenic response that establishes a limitless cell source for AC differentiation studies. A novel self-aggregation protocol was developed to maximize hyaline cartilage differentiation, and an acute joint defect model was established to evaluate the quality of engineered cartilage. Overall, these studies establish a comprehensive regenerative engineering strategy for AC that is rooted in an endogenous regenerative response.

Mammalian cartilage is not inherently regenerative so strategies to engineer cartilage have been established empirically. Historically, spontaneous chondrogenic differentiation was observed when dissociated limb bud cells are cultured at high density to mimic chondrogenic condensation (Gay and Kosher, 1984). Stem cells cultured at high density are inherently non-chondrogenic; however, chondrocytes differentiate when cultures of MSCs are treated with TGFβ1 or TGFβ3 (Johnstone et al., 1998; Mackay et al., 1998). TGFβ signaling involves binding and activation of the TGFβR1/TGFβRII receptor complex, but disruption of this signaling pathway does not influence embryonic chondrogenesis, suggesting that induced chondrogenesis by TGFβ signaling is indirect (Wang et al., 2020). TGFβR1 was found to repress formation of the high-affinity receptor complex for BMP9, Alk1/ActRIIb (Townson et al., 2012), thus implicating BMP9 signaling in embryonic chondrogenesis (Wang et al., 2019). The current findings coupled with previous findings that BMP9 induces chondrogenesis in a number of different cell culture models (Cheng et al., 2016; Majumdar et al., 2001; Morgan et al., 2020; Seemann et al., 2009) support the conclusion that BMP9 is a highly effective inducer of chondrogenesis, particularly of articular chondrocytes.

The BMP9-mediated chondrogenic response of P3 fibroblasts is rapid and robust, and represents an empirical assay, both *in vivo* and *in vitro*, for chondroprogenitor cells. Direct application of BMP9 to the uninjured digit fails to elicit a chondrogenic response, indicating an absence of BMP9-responsive chondroprogenitor cells. The chondrogenic response of P3 fibroblasts *in vitro* suggests that the sourcing of cells for culture transitions them from a non-chondroprogenitor state to chondroprogenitor cells, and a similar transition occurs during the healing response following digit amputation. This suggests that sourcing of fibroblasts for culture mimics the amputation healing response *in vivo*: both acquire chondroprogenitor characteristics that are absent in the uninjured digit. What does cell sourcing and amputation wound healing have in common that might be responsible for transitioning chondrogenic potential of fibroblasts? The answer to this question has implications for regenerative engineering in general, as many strategies begin with the isolation and expansion of a cell source. One potential answer to this question centers around enzymatic digestion of tissues to release individual cells for culture. An *in vivo* equivalent is the histolytic response of tissues undergoing regenerative and non-regenerative wound healing following digit amputation (Dawson et al., 2016; Fernando et al., 2011), which correlates with amputation-enhanced expression of ECM degrading enzymes, e.g. matrix metalloproteases (Johnson et al., 2020; Storer et al., 2020). This suggests that enzymatic ECM digestion can serve as a general activator of latent progenitor cell characteristics that are otherwise masked in uninjured tissues.

The technique of centrifugation to create 3-D high-density cell cultures that initiate chondrogenesis is widely used; however, the chondrogenic response is attenuated in central regions of the pellet that become necrotic. Central necrosis is likely attributed to reduced oxygen and/or nutrient availability. As a tissue, cartilage is avascular so chondrocytes are expected to be adaptive to hypoxic conditions (Anderson et al., 2018); however, progenitor cells prior to induction are potentially sensitive to hypoxic conditions. The strategy of self-aggregation was employed to encourage cells to establish high density culture conditions progressively and this protocol was found to prevent regional necrosis, while allowing cultures to enlarge to sizes comparable with cell pellets. BMP9 treatment of cell aggregates stimulates chondrogenic gene expression within 24 h, indicating a rapid onset of chondrogenesis and, under conditions of continuous BMP9 treatment, that aggregate size progressively increases along with a quantitative enhancement of chondrogenic genes expression. The increase in aggregate size is, in part, due to chondrocyte proliferation, as there is clear histological evidence of isogenous chondrocyte groups within P3-BMP9 engineered hyaline cartilage (see Fig. 3G). Self-aggregation represents a simple culture model to explore mechanisms guiding the growth and differentiation of hyaline cartilage, and has the potential for engineering tissue to sizes that are clinically relevant.

Given the clinical importance of joint disabilities and the potential for cell-based regenerative strategies, there are few transplantation models that allow for crucial evaluation of transplanted tissue survival, stability and integration with injured host tissues. Ectopic implantation of engineered cartilage demonstrates graft survival and stability (Craft et al., 2015; Hiramatsu et al., 2011), but not tissue integration or the influence host tissues. Large animal models are clinically relevant (Frisbie et al., 2015), but reduced sample size and a lack of cell lineage markers compromise detailed assessment of transplanted tissue. We have developed an acute MtP joint defect in immunodeficient hosts, coupled with genetically labeling (GFP) of engineered cartilage for accessing graft survival, stability, integration and injury site effects. Following engraftment, engineered hyaline cartilage does not mature into AC, indicating a need to improve articular chondrocyte differentiation prior to engraftment. Engineered hyaline cartilage integrate with host bone but there is little integration with host articular cartilage, consistent with previous studies (Bhumiratana et al., 2014). Differentiation of hypertrophic chondrocytes indicate that the injury site can be detrimental to the long-term stability of engrafted cells. Future studies are required to determine whether the hypertrophic chondrocytes are derived from hyaline chondrocytes versus undifferentiated chondroprogenitor cells of the engineered cartilage. Resolving this issue will be important for understanding the pathological progression of transplanted cartilage. As digit joints are not predicted to be subjected to significant mechanical load, the variability of the hypertrophic chondrocyte response may reflect the importance of load for osteophyte development following acute joint damage. The MtP joint injury model represents a simple and economical way to assess and improve engraftment outcomes of future regenerative engineering strategies.

## MATERIALS AND METHODS

### Animals and surgical procedures

Mouse strains used in this study included outbred CD1 purchased from Harlan Laboratories, C57BL/6-Tg(ACTBEGFP) 1Osb/J (EGFP) and NOD.CB17-Prkdcscid/J (SCID-NOD) mice purchased from Jackson Laboratories. All mice were bred in house at the Texas Institute of Genomic Medicine. All digit amputations were carried out on hindlimbs of postnatal day 3 neonatal digits of each hindlimb at the level of the second phalangeal element (P2) as previously described (Yu et al., 2019, 2012) and are referred to as digit amputation. Amputations at this level are non-regenerative and complete wound closure occurs within 4 days (Yu et al., 2012). CD1 neonates were used to isolate wound cells after the completion of wound closure following hindlimb P2-level digit amputation. EGFP mice were used to generate *Egfp* expressing fibroblasts from isolated third phalangeal elements (P3) of adult hindlimb digits to establish the P3 fibroblast cell line (*Egfp-*P3 fibroblasts). SCID-NOD mice were used as hosts for transplantation studies of engineered cartilage into an acute defect of the metatarsal-phalangeal (MtP) joint.

The MtP joint defect was surgically created in adult SCID-NOD mice. Mice were anesthetized and maintained with isoflurane (1-5% in oxygen), and buprenorphine (0.1 mg/kg) was used as a systemic analgesic. A tourniquet was placed on the hindlimb to minimize bleeding. Under a dissection microscope, the MtP joint was contracted ventrally and a 2-3 mm longitudinal skin incision was made to expose the joint capsule. A dorsal incision of the joint capsule allowed access to the proximal joint surface of the first phalangeal element (P1). An acute defect of ∼0.5 mm diameter was created in the P1 joint surface with a scalpel (Type 11, EXELINT) at the central distal groove of MtP joint. The defect extended through the articular cartilage layer and subchondral bone into the P1 bone marrow. The acute defect was cleared of residual debris by flushing with PBS prior to tissue implantation. Samples to be implanted were prepared in advance to approximately the size of the MtP defect and maintained on ice. Unused samples were processed for histological analysis to validate the cartilage phenotype. Chondrogenic samples are hard and can be compressed to fit snuggly into the acute wound site. The surface of the implant was aligned with the surface of the P1 joint and straightening of the digit maintained the positioning of the implant. The joint capsule and the overlying skin were closed with 10.0 suture (Ethicon). All animals and techniques used are compliant with the standard operating procedures and approved by the Institutional Animal Care and Use Committees at the College of Veterinary Medicine and Biomedical Sciences at Texas A&M University.

### Primary cultured amputation wound cells and P3 fibroblasts

Wound mesenchymal cells were isolated from non-regenerative digit amputation wounds following previously published protocols (Lee et al., 2013; Wu et al., 2013). Briefly, neonatal postnatal day 3 hindlimb digits were amputated at a mid-phalangeal level of P2 and wound tissue was isolated on postnatal day 7 when wound closure was complete. Each primary culture was derived from amputation wound tissue collected from 18 amputated digits. The tissue was triturated to separate connective tissue from the epidermis and the epidermis was manually removed. Approximately 1 mm of the wound was isolated in dissection medium (DMEM supplemented with 2 mM glutamine, 0.05 mg/ml gentamycin and 2% FBS) and digested in dissection medium containing 1.24U-2 U/ml liberase blendenzyme (Roche, 5401054001) for 3 h at 37°C. 10% FBS was added to quench enzymatic activity and isolated cells were washed twice with PBS prior to plating onto a 10 cm cell culture dish coated with fibronectin (2-5 µg/cm^2^). Attached amputation wound cells were maintained in 2% FBS MSC medium supplemented with EGF, PDGF and LIF, as described previously (Wu et al., 2013). The initial isolation and expansion of wound cells was designated passage 0 and all experiments were carried out with cells from passage 1 or 2. A total of 20 primary amputation wound cell cultures were used in this study. To detect cellular senescence, senescence-associated β-galactosidase activity was assessed using a commercially available kit (BioVision, #K320) following the manufacturer's instructions. *LacZ*-expressing P3 fibroblasts (*LacZ*-P3 fibroblasts) have been generated previously, and the *Egfp-*P3 fibroblast line was generated from adult mice following an identical protocol (Wu et al., 2013).

### Chondrogenic differentiation *in vitro*

Differentiation of amputation wound cells or P3 fibroblasts into hyaline cartilage was accomplished by treatment of centrifuged cell pellets or self-aggregated cell clusters in 2% FBS MSC medium supplemented with only BMP9 (100 ng/ml, R&D). Cell pellets were created by suspending 2.5×10^5^ cells in 0.5 ml of 2% FBS MSC medium in a 15 ml polypropylene culture tube and centrifuging at 150 ***g*** for 5 min at room temperature. Pellets were cultured with their caps loosened. As cultured cell pellets frequently result in necrosis of cells in the center of the pellet in P3 fibroblasts, an alternative differentiation assay involving self-aggregation of cells was developed for P3 fibroblasts. Self-aggregation was accomplished by plating 4×10^5^ cells onto Petri dishes in 2% FBS MSC medium to minimize substrate attachment for 4 days. Under these conditions, some cells form suspended aggregates that increase in size and fuse with one another with extended culture time. Media changes were carried out every 3-4 days for both differentiation assays and control cultures were treated identically but lacked BMP9 treatment.

### Histology and immunochemistry

*In vitro* differentiated tissues were fixed with Z-fix (Anatech 6269) followed by Decalcifier I (Surgipath, Leica 3800400) and processed for paraffin wax-embedded histology and immunohistochemistry. For histological analysis, the samples were stained with Mallory trichrome (Humason, 1962). Immunohistochemical staining for GFP, ColII, Acan, Prg4 and Cilp was carried out using heat retrieval [citrate buffer (pH 6) or ethylenediaminetetraacetic acid buffer (pH 8) at 90°C for 25 min] and antigen retrieval for ColX immunostaining used 1% hyaluronidase in PBS (Sigma-Aldrich H3506, room temperature, 30 min). Slides were treated in Protein Block Solution (Dako X0909; at room temperature for 1 h). Primary antibodies included anti-GFP (chicken polyclonal, Abcam 13970; 1:1000), anti-ColX (rabbit polyclonal, Abcam 58632; 1:500), anti-ColII (mouse monoclonal, Acris AF5710; 1:100), anti-Acan (rabbit polyclonal, EMD Millipore; AB1030; 1:300), anti-Prg4 (rabbit polyclonal, LSbio LS-B8236; 1:200) and anti-Cilp (rabbit polyclonal, Novus NBP1-81667; 1:100). Secondary antibodies included Alexa Fluor 568 goat anti-rabbit IgG (Invitrogen; A11011, 1:500), Alexa Fluor 568 goat anti-chicken IgG (Invitrogen, A11041, 1:500) or the Alexa Fluor goat anti-rabbit 488 IgG (Invitrogen, A11008, 1:500). Slides were counterstained with DAPI to label nuclei. Slides were imaged with an Olympus BX61 fluorescence deconvolution microscope using Slidebook software (Intelligent Imaging Innovations). Details of immunostaining procedures have been described previously (Dawson et al., 2019; Han et al., 2008; Yu et al., 2012).

### RNA analysis

*In vivo* microarrays were generated from 24 and 72 h BMP9- or BSA-treated amputated P2 digits (Yu et al., 2019) following a protocol previously described (Yu et al., 2014). Briefly, the wound mesenchyme between the P2 digit stump and wound epidermis was manually isolated under a dissection microscope and stored in RNAlater-ICE at −20°C for total RNA isolation. For *in vitro* microarrays of P3 fibroblast aggregates, cells were aggregated for 96 h then treated with BMP9 for 72 additional hours. Control untreated aggregates were prepared in parallel. Total RNA was extracted from *in vivo* or *in vitro* samples using the RNeasy Plus Micro Kit (Qiagen) following the manufacturer's recommended protocol. Each microarray analysis used the Agilent Mouse Gene Expression 8×60K G3 microarray format (G4852A) (Agilent Technologies) following manufacturer's recommended protocols. All analyses consisted of three independently collected BMP9-treated samples compared with three control samples. Data were obtained using the Agilent Feature Extraction software (v9.5) (Agilent Technologies) and were analyzed and normalized with the method described by Gene-Spring bioinformatics software (version 12.6). The unpaired unequal variance (Welch) *t*-test was used to determine significance. Genes identified as differentially expressed between BMP9 and untreated controls are based on greater than 1.5-fold change with *P*<0.05.

Quantitative reverse transcriptase polymerase chain reaction (qRT-PCR) was carried out in triplicate with the SuperScript III Platinum One-Step qRT-PCR Kit w/ROX on an Eppendorf Realplex machine according to the manufacturer's instructions. Total RNA extraction was carried out as described above and quantified and quality checked by using Nanodrop ratios of 260/280 and 260/310. For aggregation cultures at 14 and 36 days, samples were homogenized before RNA extraction. Applied Biosystem Taqman primer (Thermo Fisher) sets for the following cartilage-related genes are shown in Table S1. The expression levels of target genes were normalized to the housekeeping gene ribosomal protein L12 (RPL12) levels. Statistical significance was determined using a parametric unpaired *t*-test in Graphpad.

### Single-cell RNA-sequencing and data analysis

Neonatal cells from P2 non-regenerative digit amputation wounds were isolated as described above and 1×10^5^ passage 1 cells were plated in 10 cm culture dishes. Cells were collected after 24 h by trypsin digestion (4 min, 37°C), washed twice and resuspended in PBS with 0.08% BSA at concentration of 1×10^6^ cells/ml. Single-cell sample preparation was conducted according to Sample Preparation Protocol provided by 10× Genomics. Cell viability was assessed by Trypan Blue staining (0.4%) and determined to be greater than 90%. Subsequently, single-cell GEMs (gel bead in emulsion) and sequencing libraries were prepared using the 10× Genomics Chromium Controller in conjunction with the single-cell 3′ kit (v3). Cell suspensions were diluted in nuclease-free water to achieve a targeted cell count of 10,000 for each sample. cDNA synthesis, barcoding and library preparation were subsequently carried out according to the manufacturer's instructions. Libraries were sequenced in the Molecular Genomic Workspace of the Texas A&M Institute for Genome Sciences and Society (https://genomics.tamu.edu/) using a NovaSeq6000 sequencer (Illumina). For the mapping of reads to transcripts and cells, sample demultiplexing, barcode processing and unique molecular identifier (UMI), counts were recorded using the 10× Genomics pipeline CellRanger v5.0.1 with default parameters. Specifically, raw reads were demultiplexed using the pipeline command ‘cellranger mkfastq’ in conjunction with ‘bcl2fastq’ (v2.17.1.14, Illumina) to produce two fastq files: the read-1 file containing 26 bp reads, consisting of a cell barcode and a unique molecule identifier (UMI), and the read-2 file containing 96 bp reads, including cDNA sequences. Sequences were aligned to the mouse reference genome (mm10), filtered and counted using ‘cellranger count’ to generate the gene-barcode matrix. The resulting dataset generated 36,831 mean reads per cell, identified 4070 median genes/cell, had a sequencing saturation of 31.0% and greater than 96% of reads mapped to the genome.

Dimension reduction of expression matrices was performed using UMAP. Marker gene expression and cell type assignment was performed manually using the SC_SCATTER function of scGEAToolbox (Cai, 2019). Differential gene expression was performed using MAST (Finak et al., 2015). Labeled cell types were compared across experimental groups to quantify the differences in the level of expression. Genes with |log2(FC)|>0.25 and Benjamina-Hochberg FDR<0.05 were considered as differentially expressed. scRNA-seq data generated in this study have been deposited in GEO under accession number GSE185197.

### Isolation and differentiation of bone-derived mesenchymal stem cells

Bone-derived mesenchymal stem cells (bone MSC) were isolated and cultivated as described previously (Klepsch et al., 2013). Briefly, femurs and tibias from 6- to 8-week-old C57BL/6N mice were extracted. After flushing the bone marrow, femurs and tibias (diaphyses and epiphyses) were subjected to collagenase digestion for 3 h at 37°C, cells released from collagen-rich matrix were spun out, filtered through a cell strainer and seeded at a density of 50,000-100,000 cells/cm^2^ for propagation in bone MSC growth medium (alphaMEM supplemented with 10% FBS and penicillin/streptomycin). Cells were cultivated at 37°C in 5% CO_2_ and atmospheric oxygen. Osteogenic differentiation was either induced by 50 µg/ml ascorbic acid and 10 mM β-glycerol phosphate in growth medium (Gaddy-Kurten et al., 2002), or by StemXVivo mouse/rat osteogenic supplement (CCM009, R&D Systems) in StemXVivo adipogenic/osteogenic base media (CCM007, R&D Systems) following the manufacturer's instructions. Adipogenic differentiation was induced by incubation in StemXVivo adipogenic supplement (CCM011, R&D Systems) in base media following the manufacturer's instructions. After 21 days, mineralized matrix was detected by Alizarin Red S for osteogenic differentiation, and lipid vacuoles were visualized with Oil Red O for adipogenic differentiation. P3 fibroblasts were subjected to identical adipogenic and osteogenic differentiation regimens for 21 days.

### Flow cytometry

Flow cytometry was used to analyze P3 fibroblasts and bone MSCs. Cells were detached, filtered by 100 µm cell strainer (Corning, 431752), washed twice with flow buffer (0.5% FBS/PBS) and incubated with flow buffer at 4°C, 15 min for blocking. Cells were incubated in the dark with antibodies at a concentration of 1 µl antibody/2×10^5^ cells/100 µl at 4°C, 30 min. Cells were then washed twice and re-suspended with flow buffer and kept at 4°C in the dark prior to running flow cytometry. Flow cytometry was performed using a Gallios flow cytometer (Beckman Coulter) and flow data were analyzed using Kaluza software (Beckman Coulter). Antibodies used in this study include CD34 (48-0341-82, eBioscience), CD45 (47-0454-80, eBioscience), CD90 (47-0902-82, eBioscience), CD73 (12-0731-81, eBioscience) and CD105 (48-1051-80, eBioscience).

## Supplementary Material

Supplementary information

Reviewer comments
