# Supplementary material for: Hyaline cartilage differentiation of fibroblasts in regeneration and regenerative medicine
Source: Development. 2022 Jan 28;149(2):dev200249. doi: 10.1242/dev.200249 (PMC8917415; doi:10.1242/dev.200249)
Supplement: Supplementary information [file develop-149-200249-s1.pdf]

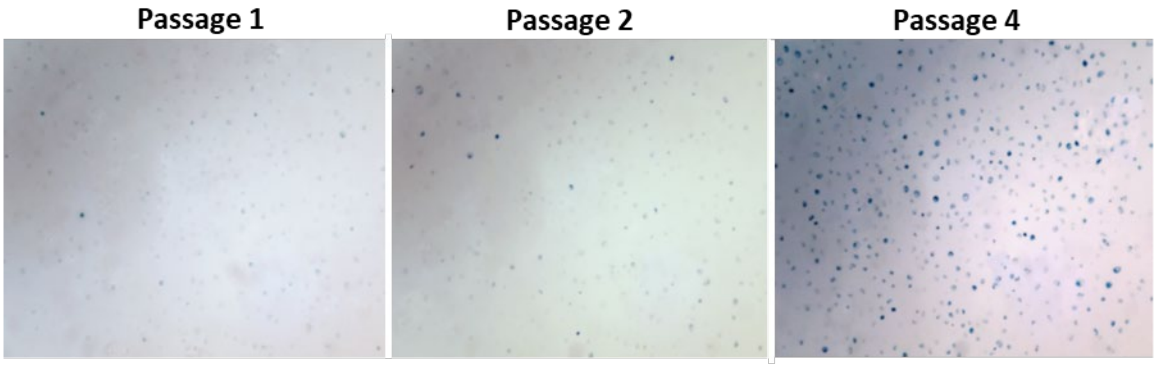

Fig. S1. Primary cultures of amputation wound mesenchymal cells derived from non-regenerative digit amputations were analyzed for senescence-associated beta-galactosidase activity at 3 different passage numbers (N=2). Senescent cells are present but not abundant after passage 1 and 2 when cells display rapid expansion. By passage 4 senescent cells are abundant and this correlates with reduced proliferation.

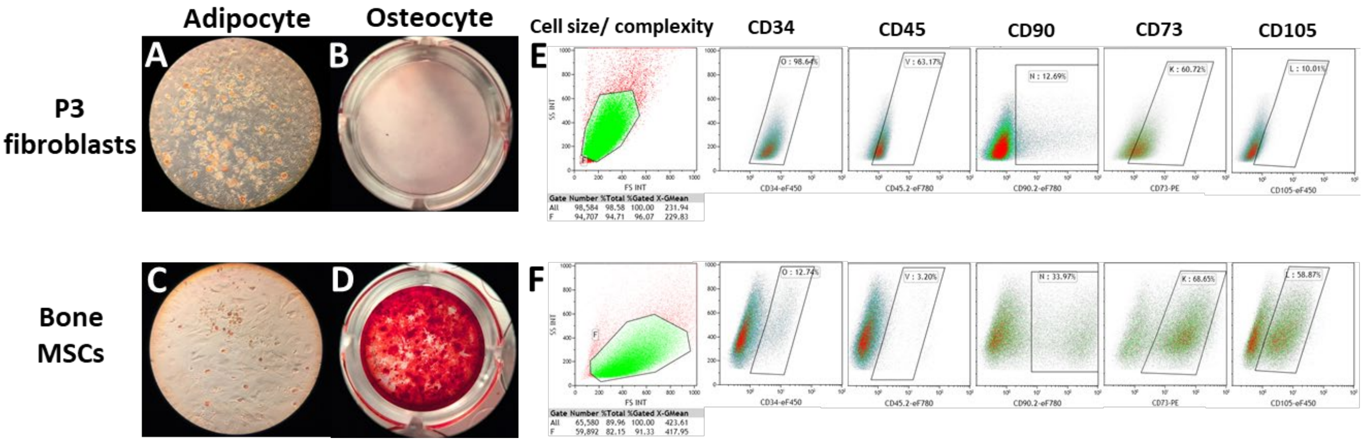

Fig. S2. Comparison of P3 fibroblasts (top) and bone MSCs (bottom) with multi-lineage differentiation ability and surface marker profiles. P3 fibroblasts can differentiate to adipocytes (A) but not osteoblasts (B). Bone MSCs, as a positive control, differentiate both adipocytes (C) and osteoblasts (D). The adipogenic differentiation was determined by Oil Red O staining and the osteogenic differentiation was identified by Alizarin Red staining. Differentiation experiments were done in triplicate wells. (E) Representative MSC surface marker phenotype by flow cytometry of P3 fibroblasts (N=2) showing that they are positive for CD73, CD34, CD45, and negative to CD90 and CD105. F) MSC surface marker phenotype of bone MSCs showing that they are positive for CD90, CD73, CD105 and negative to CD34 and CD45. The black boxes in E and F identify positive sub-populations with the percentage of cells indicated.

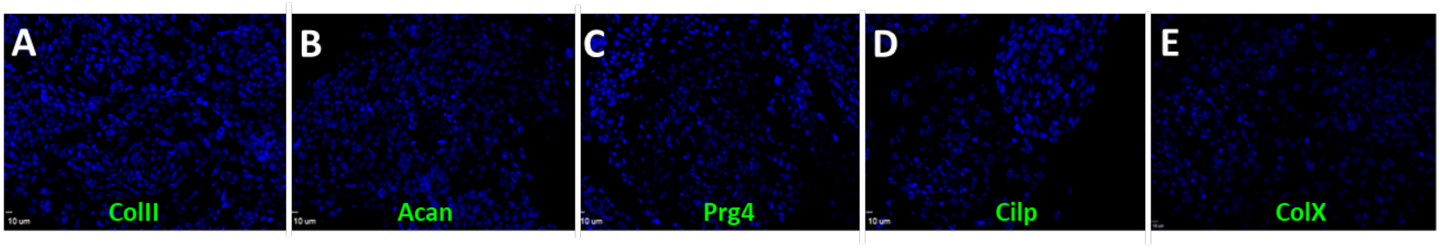

Fig. S3. Immunofluorescence staining for chondrogenic marker proteins of P3-fibroblast control (untreated) self-aggregation cultures after 36 days of culture. Control cultures are immune-negative for ColII (A), Acan (B), Prg4 (C), Cilp (D) and ColX (E) confirming histological studies showing that chondrogenesis is not stimulated in control cultures (N=2). Immuno-stained sections were counterstained with DAPI to label nuclei (blue). Scale bars A-E= 10μm

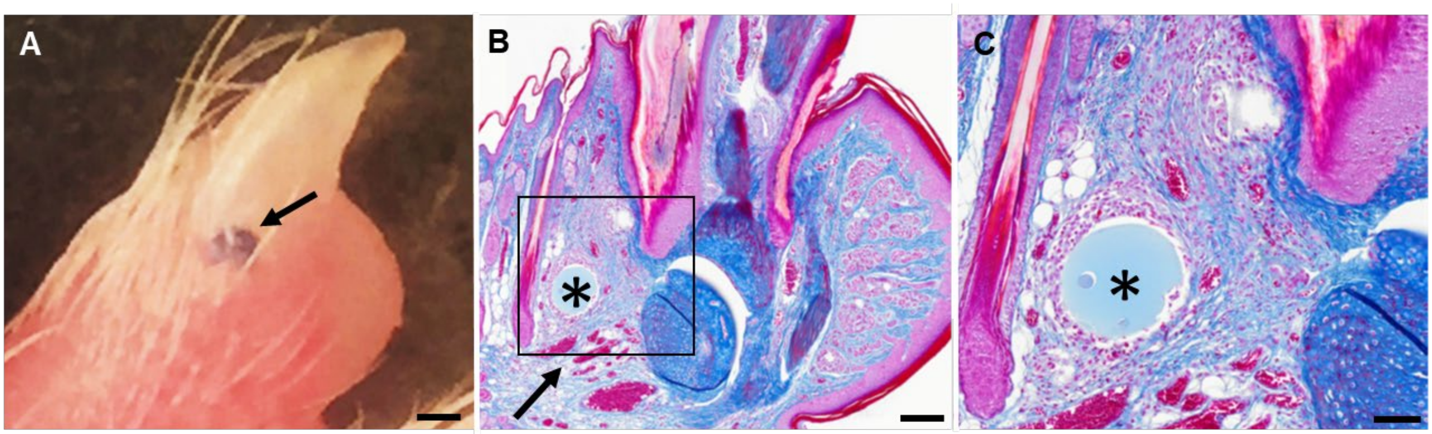

Fig. S4. To test the in vivo response to BMP9 of injured digit cells BMP9 releasing microcarrier beads (500 ng/μl) were implanted into the adult digit in proximity of the P2-P3 joint (N=6). A) External image showing an implanted BMP9 bead (arrow). B) Histological section stained with Mallory's trichome of the P2-P3 joint showing the bead (asterisk) and surrounding tissue 7 days after bead implantation. The BMP9 bead appears to elicit an angiogenic response (arrow) but no chondrocytes are induced. C) High magnification of the box in (B) showing chondrocytes of the endogenous joint but no chondrocytes associated with the BMP9 bead (asterisk). Scale bars: A,B= 200 μm, C= 50μm

Development • Supplementary information

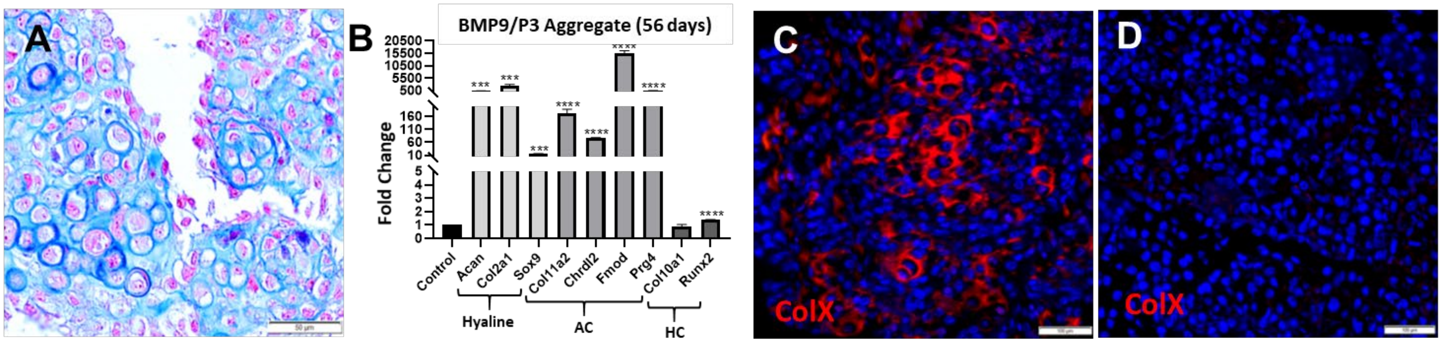

Fig. S5. A) P3 aggregates cultured with BMP9 for 56 days maintain a chondrogenic phenotype based on histological staining with Mallory's trichrome. B) qRT-PCR analysis of 56-day BMP9 treated cultures indicate that hyaline cartilage and articular cartilage specific genes are expressed at levels comparable to 36-day cultures and that hypertrophic cartilage specific genes are not minimally expressed. C) When 36-day BMP9 treated chondrogenic aggregates were treated with BMP2 instead of BMP9 for the 20 day period from 36 days to 56 days, many chondrocytes were found to be immuno-positive for ColX indicating hypertrophic chondrocyte differentiation (N=2). D) Control aggregates treated continuously with BMP9 for 56 days were immuno-negative for ColX (N=2). Immuno-stained sections were counterstained with DAPI to label nuclei (blue). Scale bar in A=50µm, C,D=100µm.

Table S1. Primer information for qRT-PCR

Name primer		Sequence information	Name primer		Sequence information
1	***Mouse Col2a1***	*Mm01309565_m1*	2	***Mouse Fmod***	Mm00491215-ml
3	***Mouse Col11a2***	Mm00487046_m1	4	***Mouse Cilp***	Mm00557687_m1
5	***Mouse Prg4***	Mm01284582_m1	6	***Mouse Scrg1***	Mm00485984_m1
7	***Mouse Acan***	Mm00545794_m1	8	***Mouse Runx2***	Mm00501584_m1
9	***Mouse Sox9***	Mm00448840_m1	10	***Mouse Col10a1***	Mm00487041_m1
11	***Mouse Chrdl2***	Mm01136674_m1	12	***Mouse Ucma***	Mm00546635_m1
13	***mouse Rpl12***	M02601627-gl			

Table S2. Cartilage-Related Gene List

Acan, Adamts12, Adamts7, Anxa6, Arid5a, Atf2, Atp6v0d2[1]*, Atp7a, Axin2, Barx2, Bbs1, Bbs2, Bgn, Bmp1, Bmp10, Bmp2, Bmp3, Bmp4, Bmp5, Bmp6, Bmp7, Bmp8a, Bmp8b, Bmpr1a, Bmpr1b, Bmpr2, Bpnt2, Carm1, Cbs, Ccl3, Ccn1, Ccn2, Ccn3, Ccn4, Cd44*[2]*, Cfh, Chadl, Chrdl2, Chst11, Chsy1, Cilp*[3]*, Clec3a, Cnmd, Col10a1, Col11a1, Col11a2, Col1a1, Col27a1, Col2a1, Col9a1, Col9a2*[4]*, Col9a3*[5]*, Comp, Cr2, Creb3l2, Crlf1*[6]*, Csgalnact1, Csgalnact2, Cst10, Ctnnb1, Ctsk, Cytl1, Dcn*[7]*, Ddrgk1, Dicer1, Dkk3, Dlk1, Dlk2, Dlx2, Dlx5*[8]*, Dspp, Ecm1, Edn1, Efemp1, Eif2ak3, Enpp2*[9]*, Ep300, Epyc, Ereg*[10]*, Erg*[11]*, Esrra, Ext1, Fam20b, Fbxw4, Fgf18, Fgf2, Fgf4, Fgf6, Fgf9, Fgfr1, Fgfr3, Fgfrl1*[12]*, Fmod*[13]*, Frzb, Fzd9*[14]*, Gata3*[15]*, Gdf2, Gdf5, Gdf6, Glg1, Gli2, Gli3, Gnas, Grem1, Gtf2ird1, Halpn1*[16]*, Hand1, Hand2, Has1*[17]*, Has2*[18]*, Hes5, Hif1a, Hmga2, Hottip, Hoxa11, Hoxa3, Hoxa5, Hoxb3, Hoxc4, Hoxd11, Hoxd3, Hspg2, Htra1, Idua, Ift80, Igfbp5*[19]*, Ihh, Il17f, Itgb8, Kat2a, Lep, Lnpk, Loxl2, Loxl3*[20]*, Lrp1*[21]*, Lrp6, Ltbp3, Maf, Mapk14, Mapk3, Matn1, Matn3, Matn4, Mboat2, Mdk, Mef2c, Mef2d, Mex3c, Mgp, Mia, Mia3, Mir140, Mir455, Mki67, Mkks, Mkx, Mmp13, Msx1, Msx2, Mustn1, Mycn, Myf5, Nfia*[22]*, Nfib, Nkx3-2, Nog, Nov*[23]*, Nppc, Opa3, Osmr, Osr1, Osr2, Otor, Pax7, Pbxip1, Pcna, Pcolce2*[24]*, Pitx1, Pkd1, Pkdcc, Poc1a, Por, Prg4, Prkca, Prkg2*[25]*, Prrx1, Prrx2, Ptger1*[26]*, Pth, Pth1r, Pthlh, Ptpn11, Rara, Rarb, Rarg, Rb1, Rbp4*[27]*, Rela, Rflna, Rflnb, Ror2, Rspo2, Runx1, Runx2, Runx3, Satb2, Scrg1*[28]*, Scube2, Scx, Sdc3*[29]*, Serpinh1, Sfrp2, Shox2, Sik3, Six2, Slc10a7, Slc29a1*[30]*, Slc39a14, Smad1, Smad3, Smad5, Smad7, Smad9, Smpd3, Snai1, Snai2, Snorc, Snx19, Sost, Sox5, Sox6, Sox9, Sprx*[31]*, Sprx2, Srf, Stc1, Stm, Sulf1, Sulf2, Tapt1, Tgfb1, Tgfb2, Tgfbr1, Tgfbr2, Thbs1, Thbs3, Thra, Thrb, Timp1, Timp2, Tnc, Trip11, Trps1, Trpv4, Ucma*[32]*, Unc5c*[33]*, Uncx, Wif1*[34]*, Wnt5a, Wnt7a, Wnt7b, Wnt9a, Wwp2*[35]*, Zbtb16, Zbtb7a, Zeb1, Zfp219, Zmpste24*

Underlined genes were added based on literature search

[1]Ayodele et al., 2017; [2]Prein and Beier, 2019; [3]Lorenzo et al., 1998; [4]Luo et al., 2017; [5]Nakayama et al., 2004; [6]Tew et al., 2007; [7]Wang et al., 2019; [8]Ferrari and Kosher, 2002; [9]Ozpolat et al., 2012; [10]Chen et al., 2018; [11]Iwamoto et al., 2013; [12]Hall et al., 2006; [13]Roughley, 2001; [14]Xu et al., 2001; [15]Singh et al., 2018; [16]Zhang et al., 2008; [17]Chan et al., 2015; [18]Matsumoto et al., 2009; [19]Brito et al., 2012; [20]Huang et al., 2016; [21]Li et al., 2020; [22]Singh et al., 2018; [23]Huang et al., 2019; [24]Steiglitz et al., 2002; [25]Koltes et al., 2015; [26]Sylvia et al., 2001; [27]Hatfield et al., 2013; [28]Ochi et al., 2006; [29]Shimazu et al., 1996; [30]Veras et al., 2019; [31]Tew et al., 2007; [32]Surmann-Schmitt et al., 2008; [33]Schubert et al., 2009; [34]Surmann-Schmitt et al., 2009; [35]Mokuda et al., 2019

Development • Supplementary information

Table S3. Microarray of BMP9 treated amputation (24h)

Click here to download Table S3

Table S4. Microarray of BMP9 treated amputation (72h)

Click here to download Table S4

Table S5. Microarray of BMP9 treated P3 fibroblast cell aggregates (72 h)

Click here to download Table S5

Ayodele, B.A., Mirams, M., Pagel, C.N., Mackie, E.J., 2017. The vacuolar H(+) ATPase V0 subunit d2 is associated with chondrocyte hypertrophy and supports chondrocyte differentiation. Bone Rep 7, 98-107.
Brito, I., Gil-Pena, H., Molinos, I., Loredo, V., Henriques-Coelho, T., Caldas-Afonso, A., Santos, F., 2012. Growth cartilage expression of growth hormone/insulin-like growth factor I axis in spontaneous and growth hormone induced catch-up growth. Growth Horm IGF Res 22, 129-133.
Chan, D.D., Xiao, W.F., Li, J., de la Motte, C.A., Sandy, J.D., Plaas, A., 2015. Deficiency of hyaluronan synthase 1 (Has1) results in chronic joint inflammation and widespread intra-articular fibrosis in a murine model of knee joint cartilage damage. Osteoarthritis Cartilage 23, 1879-1889.
Chen, Y.J., Chang, W.A., Wu, L.Y., Hsu, Y.L., Chen, C.H., Kuo, P.L., 2018. Systematic Analysis of Transcriptomic Profile of Chondrocytes in Osteoarthritic Knee Using Next-Generation Sequencing and Bioinformatics. J Clin Med 7.
Ferrari, D., Kosher, R.A., 2002. Dlx5 is a positive regulator of chondrocyte differentiation during endochondral ossification. Dev Biol 252, 257-270.
Hall, C., Flores, M.V., Murison, G., Crosier, K., Crosier, P., 2006. An essential role for zebrafish Fgfrl1 during gill cartilage development. Mech Dev 123, 925-940.
Hatfield, J.T., Anderson, P.J., Powell, B.C., 2013. Retinol-binding protein 4 is expressed in chondrocytes of developing mouse long bones: implications for a local role in formation of the secondary ossification center. Histochem Cell Biol 139, 727-734.
Huang, X., Ni, B., Mao, Z., Xi, Y., Chu, X., Zhang, R., Ma, X., You, H., 2019. NOV/CCN3 induces cartilage protection by inhibiting PI3K/AKT/mTOR pathway. J Cell Mol Med 23, 7525-7534.
Huang, Z.M., Du, S.H., Huang, L.G., Li, J.H., Xiao, L., Tong, P., 2016. Leptin promotes apoptosis and inhibits autophagy of chondrocytes through upregulating lysyl oxidase-like 3 during osteoarthritis pathogenesis. Osteoarthritis Cartilage 24, 1246-1253.
Iwamoto, M., Ohta, Y., Larmour, C., Enomoto-Iwamoto, M., 2013. Toward regeneration of articular cartilage. Birth Defects Res C Embryo Today 99, 192-202.
Koltes, J.E., Kumar, D., Kataria, R.S., Cooper, V., Reecy, J.M., 2015. Transcriptional profiling of PRKG2-null growth plate identifies putative down-stream targets of PRKG2. BMC Res Notes 8, 177.
Li, P., Fleischhauer, L., Nicolae, C., Prein, C., Farkas, Z., Saller, M.M., Prall, W.C., Wagener, R., Heilig, J., Niehoff, A., Clausen-Schaumann, H., Alberton, P., Aszodi, A., 2020. Mice Lacking the Matrilin Family of Extracellular Matrix Proteins Develop Mild Skeletal Abnormalities and Are Susceptible to Age-Associated Osteoarthritis. Int J Mol Sci 21.
Lorenzo, P., Bayliss, M.T., Heinegard, D., 1998. A novel cartilage protein (CILP) present in the mid-zone of human articular cartilage increases with age. J Biol Chem 273, 23463-23468.
Luo, Y., Sinkeviciute, D., He, Y., Karsdal, M., Henrotin, Y., Mobasheri, A., Onnerfjord, P., Bay-Jensen, A., 2017. The minor collagens in articular cartilage. Protein Cell 8, 560-572.
Matsumoto, K., Li, Y., Jakuba, C., Sugiyama, Y., Sayo, T., Okuno, M., Dealy, C.N., Toole, B.P., Takeda, J., Yamaguchi, Y., Kosher, R.A., 2009. Conditional inactivation of Has2 reveals a crucial role for hyaluronan in skeletal growth, patterning, chondrocyte maturation and joint formation in the developing limb. Development 136, 2825-2835.
Mokuda, S., Nakamichi, R., Matsuzaki, T., Ito, Y., Sato, T., Miyata, K., Inui, M., Olmer, M., Sugiyama, E., Lotz, M., Asahara, H., 2019. Wwp2 maintains cartilage homeostasis through regulation of Adamts5. Nat Commun 10, 2429.
Nakayama, N., Han, C.Y., Cam, L., Lee, J.I., Pretorius, J., Fisher, S., Rosenfeld, R., Scully, S., Nishinakamura, R., Duryea, D., Van, G., Bolon, B., Yokota, T., Zhang, K., 2004. A novel chordin-like BMP inhibitor, CHL2, expressed preferentially in chondrocytes of developing cartilage and osteoarthritic joint cartilage. Development 131, 229-240.
Ochi, K., Derfoul, A., Tuan, R.S., 2006. A predominantly articular cartilage-associated gene, SCRG1, is induced by glucocorticoid and stimulates chondrogenesis in vitro. Osteoarthritis Cartilage 14, 30-38.
Ozpolat, B.D., Zapata, M., Daniel Fruge, J., Coote, J., Lee, J., Muneoka, K., Anderson, R., 2012. Regeneration of the elbow joint in the developing chick embryo recapitulates development. Dev Biol 372, 229-238.
Prein, C., Beier, F., 2019. ECM signaling in cartilage development and endochondral ossification. Curr Top Dev Biol 133, 25-47.
Roughley, P.J., 2001. Articular cartilage and changes in arthritis: noncollagenous proteins and proteoglycans in the extracellular matrix of cartilage. Arthritis Res 3, 342-347.

Schubert, T., Denk, A., Magdefrau, U., Kaufmann, S., Bastone, P., Lowin, T., Schedel, J., Bosserhoff, A.K., 2009. Role of the netrin system of repellent factors on synovial fibroblasts in rheumatoid arthritis and osteoarthritis. Int J Immunopathol Pharmacol 22, 715-722.
Shimazu, A., Nah, H.D., Kirsch, T., Koyama, E., Leatherman, J.L., Golden, E.B., Kosher, R.A., Pacifici, M., 1996. Syndecan-3 and the control of chondrocyte proliferation during endochondral ossification. Exp Cell Res 229, 126-136.
Singh, P.N.P., Yadav, U.S., Azad, K., Goswami, P., Kinare, V., Bandyopadhyay, A., 2018. NFIA and GATA3 are crucial regulators of embryonic articular cartilage differentiation. Development 145.
Steiglitz, B.M., Keene, D.R., Greenspan, D.S., 2002. PCOLCE2 encodes a functional procollagen C-proteinase enhancer (PCPE2) that is a collagen-binding protein differing in distribution of expression and post-translational modification from the previously described PCPE1. J Biol Chem 277, 49820-49830.
Surmann-Schmitt, C., Dietz, U., Kireva, T., Adam, N., Park, J., Tagariello, A., Onnerfjord, P., Heinegard, D., Schlotzer-Schrehardt, U., Deutzmann, R., von der Mark, K., Stock, M., 2008. Ucma, a novel secreted cartilage-specific protein with implications in osteogenesis. J Biol Chem 283, 7082-7093.
Surmann-Schmitt, C., Widmann, N., Dietz, U., Saeger, B., Eitzinger, N., Nakamura, Y., Rattel, M., Latham, R., Hartmann, C., von der Mark, H., Schett, G., von der Mark, K., Stock, M., 2009. Wif-1 is expressed at cartilage-mesenchyme interfaces and impedes Wnt3a-mediated inhibition of chondrogenesis. J Cell Sci 122, 3627-3637.
Sylvia, V.L., Del Toro, F., Jr., Hardin, R.R., Dean, D.D., Boyan, B.D., Schwartz, Z., 2001. Characterization of PGE(2) receptors (EP) and their role as mediators of 1alpha,25-(OH)(2)D(3) effects on growth zone chondrocytes. J Steroid Biochem Mol Biol 78, 261-274.
Tew, S.R., Clegg, P.D., Brew, C.J., Redmond, C.M., Hardingham, T.E., 2007. SOX9 transduction of a human chondrocytic cell line identifies novel genes regulated in primary human chondrocytes and in osteoarthritis. Arthritis Res Ther 9, R107.
Veras, M.A., Tenn, N.A., Kuljanin, M., Lajoie, G.A., Hammond, J.R., Dixon, S.J., Seguin, C.A., 2019. Loss of ENT1 increases cell proliferation in the annulus fibrosus of the intervertebral disc. J Cell Physiol 234, 13705-13719.
Wang, M., Xue, S., Fang, Q., Zhang, M., He, Y., Zhang, Y., Lammi, M.J., Cao, J., Chen, J., 2019. Expression and localization of the small proteoglycans decorin and biglycan in articular cartilage of Kashin-Beck disease and rats induced by T-2 toxin and selenium deficiency. Glycoconj J 36, 451-459.
Xu, L., Tan, L., Goldring, M.B., Olsen, B.R., Li, Y., 2001. Expression of frizzled genes in mouse costochondral chondrocytes. Matrix Biol 20, 147-151.
Zhang, M., Pritchard, M.R., Middleton, F.A., Horton, J.A., Damron, T.A., 2008. Microarray analysis of perichondral and reserve growth plate zones identifies differential gene expressions and signal pathways. Bone 43, 511-520.
