# Supplementary material for: Hyaline cartilage differentiation of fibroblasts in regeneration and regenerative medicine
Source: Development. 2022 Jan 28;149(2):dev200249. doi: 10.1242/dev.200249 (PMC8917415; doi:10.1242/dev.200249)
Supplement: Reviewer comments [file develop-149-200249_review_history.pdf]

Hyaline cartilage differentiation of fibroblasts in regeneration and regenerative medicine

Ling Yu, Yu-Lieh Lin, Mingquan Yan, Tao Li, Emily Y. Wu, Katherine Zimmel, Osama Qureshi, Alyssa Falck, Kirby M. Sherman, Shannon S. Huggins, Daniel Osorio Hurtado, Larry J. Suva, Dana Gaddy, James Cai, Regina Brunauer, Lindsay A. Dawson and Ken Muneoka
DOI: 10.1242/dev.200249

Editor: James Wells

Review timeline

Original submission:	8 October 2021
Editorial decision:	9 November 2021
First revision received:	13 December 2021
Accepted:	15 December 2021

Original submission

First decision letter

MS ID#: DEVELOP/2021/200249

MS TITLE: Hyaline cartilage differentiation of fibroblasts in regeneration and regenerative medicine

AUTHORS: Ken Muneoka, Ling Yu, Yu-Lieh Lin, Mingquan Yan, Tao Li, Emily Y Wu, Katherine Zimmel, Osama Qureshi, Alyssa Falck, Kirby M Sherman, Shannon S Huggins, Daniel Osorio Hurtado, Larry J Suva, Dana Gaddy, James Cai, Regina Brunauer, and Lindsay A Dawson

I have now received all the referees reports on the above manuscript, and have reached a decision. The referees' comments are appended below, or you can access them online: please go to BenchPress and click on the 'Manuscripts with Decisions' queue in the Author Area.

The overall evaluation is largely positive and we would like to publish a revised manuscript in Development, provided that the referees' comments can be satisfactorily addressed. While some additional wet lab experiments were suggested, we do not feel that these are needed. However, the reviewers still had some valid concerns about how the manuscript is written, how some data were interpreted and explained, and that the single cell data could be more deeply analyzed and explained. Please address the comments in your revised manuscript and detail them in your point-by-point response. If you do not agree with any of their criticisms or suggestions explain clearly why this is so.

We are aware that you may currently be unable to access the lab to undertake experimental revisions. If it would be helpful, we encourage you to contact us to discuss your revision in greater detail. Please send us a point-by-point response indicating where you are able to address concerns raised (either experimentally or by changes to the text) and where you will not be able to do so within the normal timeframe of a revision. We will then provide further guidance. Please also note that we are happy to extend revision timeframes as necessary.

© 2021. Published by The Company of Biologists under the terms of the Creative Commons Attribution License (https://creativecommons.org/licenses/by/4.0/).

Reviewer 1

Advance summary and potential significance to field

Yu et al. revised their manuscript with additional data and more details about the experiments.

Comments for the author

I thank the authors for their further analysis on the presence of MSCs in their cultures, methodological details, and discussions. I still think their findings can have important implications for the field. However, the text is lengthy and confusing on its central message (as the Rev2 indicated, is this manuscript about P3 fibroblast competency, or a new grafting method etc.???), and I do not think the revised text and experiments address this point sufficiently. Moreover, the incorporation of single-cell data looks extremely premature. The current state of this data is not suitable for me to comment on the findings. Neither how it is included in the text or as a figure looks on par with any other contemporary study. Unfortunately, I cannot be positive about the publication of this manuscript.

Reviewer 2

Advance summary and potential significance to field

The authors took a very unique approach and showed that BMP9 has a strong chondrogenic effect. In addition, the authors are to be commended for presenting the potential application of the induced cartilage for joint regeneration. This is a highly original study, and it may be difficult to gain the understanding of many researchers. I believe that this is research that can present great possibilities if the authors make a little more careful explanation. I am sure that this is pioneering a new field and definitely has a great value to be published.

Comments for the author

The following is a list of possible corrections that could be made before publication.
Line 317; How did the authors determine these time points? need an explanation on this point.
Line 382; Please check the relationship between cell proliferation and cell type.
B9 is received by only chondrogenic cells or effective for only chondrogenic cells?
Line 398; Fig1A shows no sign of articulation. The authors performed the cell culture as an aggregate. thus, it would be possible to investigate gene expression patterns in the aggregate. IHSC of ISH would be the best to show gene expression patterns in aggregates.
Line 421; The determination of "fibroblasts" is ambiguous. The determination totally relies on the external standard. Scientists in other fields determine fibroblasts with their original determination. To avoid ambiguity, I offer the authors to add "AC and HC" cells in the scRNA analysis. If those appear as an isolated population, it can be determined that the cell the authors collected were distinguished cell populations from chondrocytes.
Line 453; The storyline is a little complicated for me. If I am missing something please forgive me. But, as far as I know, adult mice can regenerate their digit tips. If the authors prepare cells from the digit tip of an adult mouse, can the author see cell population just like neonate wound fibroblasts?
Line 455; Please consider calling "fibroblasts" in the manuscript. This is so biased usage of the word. How do the authors determine this? Usage of the word
“fibroblasts” might impress readers with an image of transdifferentation from fibroblasts to chondrogenic cells. However, in the present manuscript, there is no data to demonstrate “transdifferentiation”.
Fig2A; What do the authors describe the isolated population colored in yellow (the-right most population)? How to isolate this? What factors make this isolation? please describe it in the text. This is a very interesting population and should be worth noting.
Line 467; please plot the scRNA data of the P3 fibroblasts. This is ultimately necessary to know what is in the P3 cell population.
Fig. 3; The result is interesting. P3 is derived from the regenerative region in a mouse digit. And BMP2 is the regeneration inducer in P3/P2 region. In the B2 induced regeneration, cartilage cells

© 2021. Published by The Company of Biologists under the terms of the Creative Commons Attribution License (https://creativecommons.org/licenses/by/4.0/).

appear as well as osteocytes. However, the culture with B2 seems not to be consistent. Based on this, I have the same questions on the results of Fig 3. First, did B2 treatment promote osteogenesis (check ColX expression)? Second, are the authors saying P3 fibroblasts are joint cartilage-committed cell populations? Or do the authors want to insist that BMP9 promote transdifferentiation. If the P3 cells are the population that can be responsible for the digit tip regeneration, BMP2 should promoter chondrogenesis and/or osteogenesis. Or, did the authors establish an articular chondrogenic cell line by chance? In this, case, there is little meaning to use P3 cells. Rather the authors should use a cell line determined as joint cartilage cells.
Line 560-; Please clarify the determination of the integration the author call in the manuscript. As long as I saw the reimplantated part appears not to be integrated.

Line 602; Did all GFP+ cells are ACN+? please provide a higher magnification view.
Line 604; This is very impressive. I suggest the author to add B2 control in Fig 6.
Overall, I think it is important for the authors to describe P3 cells properly and to show whether P3 is a cell population destined for articular cartilage or a cell population that can differentiate into digit bone cells and cartilage cells.
Besides, the difference and linkage between P3 and the cell populations in Fig.1-2 needs to be examined a little more. The gap between them makes the manuscript difficult to understand. In addition, if BMP9 has a universal chondrogenic effect then ES, BMSCs, iPSCs, etc. can be used to induce chondrogenesis with the same results. I think it is extremely important to accurately describe here whether there is a need to use P3 or not, and if there is a need to use P3, what kind of properties the cell population has, in order to aim for future applications.

Reviewer 3

Advance summary and potential significance to field

I appreciate the effort of the authors to revise this manuscript. Overall, the additional data and clarifications raise the potential significance and impact of these studies. The manuscript provides value to the scientific community and should be published. However, my enthusiasm remains moderate as the studies do not establish the importance of Bmp9 and the value of this new wound healing model as strongly as expected for a full research article.

Comments for the author

No new comments

First revision

Author response to reviewers' comments

Detailed Response to Review Comments

Reviewer 1 Comments for the Author:
I thank the authors for their further analysis on the presence of MSCs in their cultures, methodological details, and discussions. I still think their findings can have important implications for the field. However, the text is lengthy and confusing on its central message (as the Rev2 indicated, is this manuscript about P3 fibroblast competency, or a new grafting method etc.???), and I do not think the revised text and experiments address this point sufficiently. Moreover, the incorporation of single-cell data looks extremely premature. The current state of this data is not suitable for me to comment on the findings. Neither how it is included in the text or as a figure looks on par with any other contemporary study. Unfortunately, I cannot be positive about the publication of this manuscript.

Response: We thank the reviewer for the input. The text is lengthy and complicated because the paper describes studies that bridge two fields: Regeneration Biology and Regenerative Medicine.

© 2021. Published by The Company of Biologists under the terms of the Creative Commons Attribution License (https://creativecommons.org/licenses/by/4.0/).

It is not focused on either fibroblast competency or new methods but on comparable studies that can impact both fields and we have separated the discussion to emphasize this. In the current revision, we have reduced the text considerably to be more concise and much of the newly added data was moved to the supplemental data file. We do not agree that the single-cell data is premature because it was introduced to answer an important question: what cell type is responding to BMP9. Unlike many contemporary studies that are focused entirely on single cell technology we have restricted our analysis to aspects of the dataset that relate to this specific question. After publication this dataset will be readily available for future detailed analyses to address additional questions not germane to the current manuscript.

Reviewer 2 Advance Summary and Potential Significance to Field: The authors took a very unique approach and showed that BMP9 has a strong chondrogenic effect. In addition, the authors are to be commended for presenting the potential application of the induced cartilage for joint regeneration. This is a highly original study, and it may be difficult to gain the understanding of many researchers. I believe that this is research that can present great possibilities if the authors make a little more careful explanation. I am sure that this is pioneering a new field and definitely has a great value to be published.

Response: We appreciate this comment and have tried our best to make the study understandable to other researchers.

Reviewer 2 Comments for the Author:
The following is a list of possible corrections that could be made before publication.

1) Line 317; How did the authors determine these time points? need an explanation on this point.

Response: We have added a statement indicating that the time points selected to correspond to previous in situ hybridization studies (lines 133-134; identified in yellow).

2) Line 382; Please check the relationship between cell proliferation and cell type. B9 is received by only chondrogenic cells or effective for only chondrogenic cells?

Response: We did not analyze the proliferative response of chondrocytes versus non-chondrocytes in this study. In vivo cell proliferation studies (Yu et al., 2019) indicated that proliferation is not stimulated in BMP9 induced chondrocytes. BMP9 stimulates wound cells isolated from the amputation wound but these cells are not chondrogenic based on scRNAseq analysis, they are fibroblastic. The data indicate that BMP9 stimulates fibroblasts to become chondroctyes.

3) Line 398; Fig1A shows no sign of articulation. The authors performed the cell culture as an aggregate. thus, it would be possible to investigate gene expression patterns in the aggregate. IHSC of ISH would be the best to show gene expression patterns in aggregates.

Response: The reviewer is correct that there is no indication of a skeletal articulation in cultures of cell aggregates (pellet or self-aggregation). Instead, the histology shows that cell aggregates differentiate as uniform cartilage that lacks evidence of organization into a skeletal articulation as observed in vivo. We do provide IHSC in Fig 3H-I that shows uniform expression of ColII and Acan for BMP9 induced P3 cells that and confirms the lack of articulation. Replication of IHSC for ampWMCs is redundant given the histological evidence.

4) Line 421; The determination of "fibroblasts" is ambiguous. The determination totally relies on the external standard. Scientists in other fields determine fibroblasts with their original determination. To avoid ambiguity, I offer the authors to add "AC and HC" cells in the scRNA analysis. If those appear as an isolated population, it can be determined that the cell the authors collected were distinguished cell populations from chondrocytes.

Response: We respectfully disagree with this assessment. Fibroblast determination was carried out using an unbiased online analytical tool (PanglaoDB) that established a community-curated cell-type marker compendium as a resource for automatic annotation of cell types. We supplemented the PanglaoDB determination of fibroblasts with targeted analyses of limb and tissue-type specific fibroblast cell markers from recent publications to compare wound fibroblast

© 2021. Published by The Company of Biologists under the terms of the Creative Commons Attribution License (https://creativecommons.org/licenses/by/4.0/).

in situ to cultured wound cells. We have modified the text to emphasize the PanglaoDB component of the analysis (lines 198-200, marked in yellow). Since the PangoaoDB assessment includes chondrocytes, the comparison to AC and HC is a component of our analysis.

5) Line 453; The storyline is a little complicated for me. If I am missing something, please forgive me. But, as far as I know, adult mice can regenerate their digit tips. If the authors prepare cells from the digit tip of an adult mouse, can the author see cell population just like neonate wound fibroblasts?

Response: The reviewer is correct that the adult digit tip of mice can regenerate however; in this study we did not compare fibroblasts from regenerating digit tips. Since ampWMC are derived from non-regenerating digit tip amputations (i.e. amputations proximal to the digit tip at the level of the second phalangeal element (P2)) we compared fibroblasts from non-regenerating amputation wounds. Based on scRNAseq mapping we did not observe neonatal fibroblasts that overlapped with adult amputation wound fibroblasts.

6) Line 455; Please consider calling "fibroblasts" in the manuscript. This is so biased usage of the word. How do the authors determine this? Usage of the word “fibroblasts” might impress readers with an image of transdifferentation from fibroblasts to chondrogenic cells. However, in the present manuscript, there is no data to demonstrate “transdifferentiation”.

Response: We understand the potential for confusion with respect to transdifferentiation during regeneration. However, many studies show that chondrocytes differentiate from progenitor cells that display fibroblastic characteristics and scRNAseq data identifies these cells as fibroblasts. After BMP9 treatment, these cells differentiate to chondrocytes. Use of the term fibroblasts in this study is justified and best described the cells type responsive to BMP9. The reviewer is correct that there are no data demonstrating transdifferentiation and we do not address or discuss the transdifferentiation issue in this paper.

7) Fig2A; What do the authors describe the isolated population colored in yellow (the-right most population)? How to isolate this? What factors make this isolation? please describe it in the text. This is a very interesting population and should be worth noting.

Response: The cells are a subpopulation of amputation wound cells that display a transcriptome distinct from the larger population of amputation wound cells when projected with adult amputation wound cells. We agree that these cells are potentially interesting as are other distinct fibroblast cell clusters that have been reported in recent scRNAseq studies on regeneration. The purpose for turning to scRNAseq for this study was to address the question of cell type and we remain focused on that purpose. It is clear that scRNAseq datasets offer a wealth of information for future studies and once this dataset is made public it will be available for anyone to explore. At this time we are unable to provide answers to the very interesting questions posed however these questions are beyond the scope of the current manuscript.

8) Line 467; please plot the scRNA data of the P3 fibroblasts. This is ultimately necessary to know what is in the P3 cell population.

Response: scRNAseq was used in this study was to determine the cell type of BMP9 responsive wound cells as fibroblasts. The fibroblastic nature of P3 cells were previously published (PMID: 23349966), and unpublished scRNAseq dataset confirms this. The scRNAseq dataset for P3 fibroblasts is currently being analyzed in more detail and will be published at a later date.

9) Fig. 3; The result is interesting. P3 is derived from the regenerative region in a mouse digit. And BMP2 is the regeneration inducer in P3/P2 region. In the B2 induced regeneration, cartilage cells appear as well as osteocytes. However, the culture with B2 seems not to be consistent. Based on this, I have the same questions on the results of Fig 3. First, did B2 treatment promote osteogenesis (check ColX expression)? Second, are the authors saying P3 fibroblasts are joint cartilage-committed cell populations? Or do the authors want to insist that BMP9 promote transdifferentiation. If the P3 cells are the population that can be responsible for the digit tip regeneration, BMP2 should promoter chondrogenesis and/or osteogenesis. Or, did the authors establish an articular chondrogenic cell line by chance? In this, case, there is

© 2021. Published by The Company of Biologists under the terms of the Creative Commons Attribution License (https://creativecommons.org/licenses/by/4.0/).

little meaning to use P3 cells. Rather, the authors should use a cell line determined as joint cartilage cells.

Response: First, BMP2 treatment of P3 aggregates does not promote chondrogenesis or osteogenesis as shown in figure 3E. However, after BMP9 treatment to induce chondrogenesis, subsequent treatment with BMP2 induces ColX expression as shown in Supplemental figure 5 indicating a BMP2 link to osteogenesis. At this time, we do not know if the cells responding to BMP2 are the same as those responding to BMP9, so the inconsistency of the in vivo and in vitro BMP2 response might suggest that they are different. The response to BMP2 is of interest but beyond the scope of the current study. Second, we do not conclude that BMP9 promotes transdifferentiation but that the cell line contains chondroprogenitor cells that possess the potential to differentiate hyaline cartilage. In a previous paper (PMID: 23349966) we determined that P3 fibroblasts can contribute to blastema formation and digit tip regeneration but these cells are not responsible for stimulating regeneration. We do not conclude that P3 cells are responsible for digit tip regeneration, only that they represent a cell line that displays a chondrogenic response to BMP9. Third, searched for a chondroprogenitor cell based on the evolutionary argument that regenerative failure in mammals was lost and could be re-gained, so these studies are not based on a “chance” discovery. We are not aware of any articular cartilage cell lines and we believe that identifying one represents an important advance for studying articular cartilage regeneration, both in the context of regeneration and regenerative medicine.

10) Line 560-; Please clarify the determination of the integration the author call in the manuscript. As long as I saw the reimplantated part appears not to be integrated.

Response: We apologize for the confusion. The question of implant integration is addressed in histological images shown in Figure 6E and 6I. We have changed the language and how describe regions of the implant as tightly adhering to host bone tissue versus not adhering to articular cartilage tissue (Lines 335-336 marked in yellow). For clarity, we have indicated local regions of non-adherence in figure 6E and with an *.

11) Line 602; Did all GFP+ cells are ACN+? please provide a higher magnification view.

Response: We did not observe 100% correspondence between GFP+ and Acan+ cells and have modified the text to reflect this (line 344 marked in yellow). Images are shown at a low magnification to emphasize that co-expressing cells are found throughout the implant and that Acan expression in contiguous across host and implanted tissue. High resolution insets of cell clusters have been added to Figure 6H and L to show examples of co-expressing cells. We have added arrows to Figure 6F,G,J,K to identify the cell clusters in low magnification images for GFP and Acan, and the figure legend has been modified (lines 762-764 marked in yellow, and 768-771 marked in yellow).

12) Line 604; This is very impressive. I suggest the author to add B2 control in Fig 6

Response: The appropriate implant control for BMP9 treatment is the vehicle control without BMP9. BMP2 is not a control but a separate experimental study. BMP2 does not induce chondrogenesis as shown in Figure 3E so there is no reason think BMP2 treatment would differ from the vehicle control that fails to undergo chondrogenesis. Adding BMP2 to this study is unreasonable, would add little to the study and represents an unnecessary delay in publication.

13) Overall, I think it is important for the authors to describe P3 cells properly, and to show whether P3 is a cell population destined for articular cartilage or a cell population that can differentiate into digit bone cells and cartilage cells. Besides, the difference and linkage between P3 and the cell populations in Fig.1-2 needs to be examined a little more. The gap between them makes the manuscript difficult to understand. In addition, if BMP9 has a universal chondrogenic effect, then ES, BMSCs, iPSCs, etc. can be used to induce chondrogenesis with the same results. I think it is extremely important to accurately describe here whether there is a need to use P3 or not, and if there is a need to use P3, what kind of properties the cell population has, in order to aim for future applications.

© 2021. Published by The Company of Biologists under the terms of the Creative Commons Attribution License (https://creativecommons.org/licenses/by/4.0/).

Response: We believe we have described P3 cells properly and accurately. P3 cells contain chondroprogenitors because they can differentiate into hyaline cartilage that contain articular chondrocytes (Figure 3) and hypertrophic chondrocytes (Figure 6N, O and Supplemental Figure 5) but not osteoblasts (Supplemental Figure 2). We are careful not to conclude they are not destined for articular cartilage but can be directed toward articular cartilage differentiation by BMP9. We do not promote the idea that BMP9 has a universal chondrogenic effect, indeed there is considerable evidence that BMP9 influence is cell type specific.

One of the challenges for the field of Regenerative Biology is to make inroads into understanding why regeneration is restricted in mammals, particularly humans, and how regenerative capabilities can be expanded. Alternatively, the field of Regenerative Medicine has a strong stem cell focus and takes an engineering approach to solving problems associated with regenerative failure without addressing the question of why regeneration failed. We see the similarities between the BMP9 response of amputation wound cells and P3 cells as a bridge between two areas of research, and we agree that the difference and linkage between them needs further examination. In this initial manuscript our goal is to bring attention to the similarities between the in vivo versus in vitro BMP9 response and to establish a model to study Regeneration in the context of Regenerative Medicine. Because this is a novel approach and the first of its kind, we accept that manuscript may be difficult to understand and will get some resistance. We purposefully separated the discussion into a Regeneration Biology section and a Regenerative Medicine section to emphasize how the studies impact both fields.

Reviewer 3 Advance Summary and Potential Significance to Field:
I appreciate the effort of the authors to revise this manuscript. Overall, the additional data and clarifications raise the potential significance and impact of these studies. The manuscript provides value to the scientific community and should be published. However, my enthusiasm remains moderate as the studies do not establish the importance of Bmp9 and the value of this new wound healing model as strongly as expected for a full research article.
Response: We appreciate and accept the reviewer’s point of view.

<u>Second decision letter</u>

MS ID#: DEVELOP/2021/200249

MS TITLE: Hyaline cartilage differentiation of fibroblasts in regeneration and regenerative medicine

AUTHORS: Ling Yu, Yu-Lieh Lin, Mingquan Yan, Tao Li, Emily Y Wu, Katherine Zimmel, Osama Qureshi, Alyssa Falck, Kirby M Sherman, Shannon S Huggins, Daniel Osorio Hurtado, Larry J Suva, Dana Gaddy, James Cai, Regina Brunauer, Lindsay A Dawson, and Ken Muneoka
ARTICLE TYPE: Research Article

I am happy to tell you that your manuscript has been accepted for publication in Development, pending our standard ethics checks. We appreciate the efforts that the authors have put into this revised manuscript and believe that it is an important advance. In particular, the single cell data comparing adult vs neonatal cells indicates that there are very different populations of ‘fibroblasts’¤. The adult has three distinct populations and neonate has two. Both reviewers 1 and 2 asked for more granularity about what distinguishes different populations of ‘fibroblasts’. While your manuscript has been accepted, I encourage you to consider modifying figure 2 to better highlight some of those differences. Currently figure 2 highlights a handful of markers that are broadly expressed in fibroblasts. Showing examples of markers that distinguish the different fibroblast populations in figure 2, along with a table that highlight the transcriptional differences that are driving the separation of fibroblast populations during clustering, should be very simple, address the reviewers’ comments, and provide insight into the biology of the system. This is your decision and just a suggestion on my part.

© 2021. Published by The Company of Biologists under the terms of the Creative Commons Attribution License (https://creativecommons.org/licenses/by/4.0/).
